# You Don’t Learn That in School: An Updated Practical Guide to Carbon Quantum Dots

**DOI:** 10.3390/nano11030611

**Published:** 2021-03-01

**Authors:** Helena B. A. Sousa, Catarina S. M. Martins, João A. V. Prior

**Affiliations:** LAQV, REQUIMTE, Laboratory of Applied Chemistry, Department of Chemical Sciences, Faculty of Pharmacy, University of Porto, Rua de Jorge Viterbo Ferreira n. 228, 4050-313 Porto, Portugal; hbffup@gmail.com (H.B.A.S.); catsofiamartins@gmail.com (C.S.M.M.)

**Keywords:** carbon quantum dots, synthesis, surface modifications, separation methods, chemosensors, biosensors

## Abstract

Carbon quantum dots (CQDs) have started to emerge as candidates for application in cell imaging, biosensing, and targeted drug delivery, amongst other research fields, due to their unique properties. Those applications are possible as the CQDs exhibit tunable fluorescence, biocompatibility, and a versatile surface. This review aims to summarize the recent development in the field of CQDs research, namely the latest synthesis progress concerning materials/methods, surface modifications, characterization methods, and purification techniques. Furthermore, this work will systematically explore the several applications CQDs have been subjected to, such as bioimaging, fluorescence sensing, and cancer/gene therapy. Finally, we will briefly discuss in the concluding section the present and future challenges, as well as future perspectives and views regarding the emerging paradigm that is the CQDs research field.

## 1. Introduction

Carbon quantum dots (CQDs) are a type of zero-dimensional carbonaceous fluorescent nanomaterial, generally smaller than 10 nm. Structurally, CQDs are clusters of carbon atoms with a substantial percentage of oxygen and hydrogen atoms at their surface, resulting in low toxicity and excellent biocompatibility that make CQDs promising fluorescent platforms for numerous applications, particularly when compared with conventional metal quantum dots [1,2,3]. CQDs were serendipitously discovered in 2004 by Xu et al. during the processing of single-walled carbon nanotubes (SWCNT) through gel electrophoresis [4].

Presently, CQDs are often mistaken for their carbon-based counterparts, such as graphene quantum dots (GQDs) or carbon nanodots (CNDs), despite the strikingly dissimilar properties, as depicted in Table 1. Based in the systematic classification employed by Valcárcel et al. [3], CQDs correspond to crystalline spherical structures with quantum confinement composed mainly by a mixture of sp^2^ and sp^3^ carbons, and GQDs are composed by single nanosheets of sp^2^ carbons, whereas CNDs can be described as quasi-spherical amalgams of chiefly sp^3^ carbons in an amorphous structure that lacks quantum confinement. Additionally, the observation of CQDs via HRTEM reveals lattice fringes of 0.34 nm matching the (002) interlayer spacing of graphite, while GQDs present lattice fringes of 0.24 nm consistent with the (100) interlayer spacing of graphite [5,6]. Through the variation of the synthesis precursors and methods used during their production, it is possible to obtain these distinct types of carbonaceous nanomaterials.

Due to their structure, CQDs are known to possess important features that have, over the years, made them attractive for usage in photomediated applications. Amongst their many qualities, CQDs display tunable photoluminescence (PL), biocompatibility, broad emission range, high photostability, and they can behave either as electron donors or acceptors [6].

**Figure 1:**
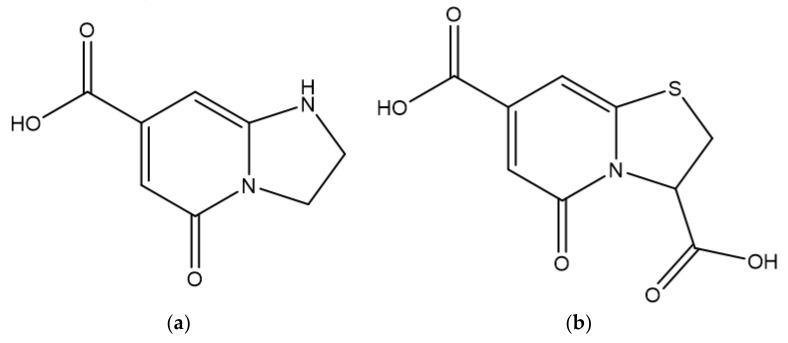
Chemical structures of IPCA (**a**) and TPDCA (**b**).

As for the fluorescence mechanism that grants CQDs with their unique optical properties, it is yet to be completely clarified. However, three possible mechanisms have been proposed: quantum confinement effect, surface defect states, and incorporation of fluorophores. Quantum confinement effect (QCE) is a phenomenon that occurs when CQDs display a size smaller than the exciton Bohr radius. The smaller the CQDs, the higher the band gap energy between the valence band and conduction band, which results in a blue-shift in CQDs photoluminescence. This causes a band gap transition in the ultraviolet-visible region. As the optical properties of CQDs are directly linked to the π-electron state of the sp^2^ carbons, modifying the size of the conjugated π-domains will result in either a promotion or inhibition of the direct transition of conduction band electrons to the valence band, which is the process responsible for band gap fluorescence [7,8]. Other than quantum confinement effect, surface defect states have also been pointed out as a possible fluorescence mechanism. Surface defects correspond to a spheroidal area separate from CQDs’ carbon core, mainly originated due to surface oxidation. By acting as capture centers for excitons, surface defects generally lead to a multicolor light emission, being responsible for excitation dependent behavior. Higher amounts of surface defects, corresponding to a greater degree of surface oxidation, generate a red-shift in the emission wavelength. Finally, the presence of fluorophores is also a common cause for CQDs fluorescence, especially in synthesis using smaller molecules as starting materials [7]. Due to being low molecular weight molecules, during the synthesis process they easily establish connections that may originate fluorophores with aromatic structures such as 1,2,3,5-tetrahydro-5-oxo-imidazo [1,2-a]pyridine-7-carboxylic acid (IPCA) or 5-oxo-3,5-dihydro-2H-thiazolo [3,2-a] pyridine-3,7-dicarboxylic acid (TPDCA), both depicted in Figure 1.

Due to recent developments in the purification area, there has been an increasingly clear relationship between the quantum yield of CQDs and the presence of these fluorophores both on the surface of CQDs and in solution. As they are water-soluble, a good purification method is important to guarantee that the determined quantum yield corresponds to CQDs and not to fluorophores.

Other than their exquisite optical properties, CQDs present higher chemical and physical stability and lower cost than conventional fluorescent nanoparticles, namely semiconductor quantum dots. Additionally, and of most importance, their outstanding resistance to photobleaching is a great advantage over the semiconductor quantum dots or organic dyes, since CQDs are much less susceptible to light exposure, especially when well dispersed. Furthermore, stabilizing CQDs in a polymer matrix can further contribute to preserving their characteristic fluorescence [9,10]. These properties coupled with good cell permeability, weak interactions with serum proteins, as well as immune system evasion, have made CQDs the ideal fluorescent nanosystem in applications that range from bioimaging, optoelectronics, energy conversion, and gene/drug delivery to their usage as antimicrobial agents or in chemical sensing [1,6,11,12,13,14,15]. Moreover, several works have focused specifically on the ecologically sustainable synthesis of CQDs, and as such, their applications in previously unexplored settings are expected to spread out even further in the impending future [12,16,17] To efficiently tune the intrinsic optic and electron transfer properties of CQDs, specific synthesis strategies must be adopted to modulate their size and surface configuration, namely usage of distinct synthesis routes, surface passivation, and/or functionalization or heteroatom doping [15,18].

In the present review, a systematic and concise approach has been taken to survey the latest state of the art (between 2018 and 2020) of CQDs in terms of synthesis, concerning both the methodologies and the starting materials employed. Additionally, this work will emphasize the importance of adequate characterization and purification methods and their impact in the final quality of CQDs. Several surface modifications and heteroatom doping may be carried out to optimize CQDs properties to ensure their best performance in multiple applications, and as such, those modifications are likewise a feature in the present work. The diverse applications in which the CQDs are used will be explored with the aim of improving the current insights into the importance and potential of CQDs in fields as diverse as cell imaging, biosensing, or targeted drug delivery. Finally, a brief overview of the difficulties and challenges presented nowadays in CQDs synthesis and usage is provided, to explore possible future directions and perspectives in the field of CQDs research.

## 2. Synthesis Methods of CQDs

Typically, the strategies of CQDs synthesis are organized in two main categories, top-down and bottom-up routes. While the top-down route requires rupturing or splitting more complex molecular structures into smaller ones through physical, chemical, or electrochemical methods, CQDs prepared by the bottom-up route are formed either by carbonization or pyrolysis of small organic precursors into nanosized particles. These organic molecules generally undertake four stages during the growth of CQDs, namely condensation, polymerization, carbonization, and passivation [6,13,19]. Commonly used top-down approaches include electrochemical oxidation (electrolysis), laser ablation, sonochemical synthesis, and arc-discharge methods, whereas bottom-up approaches usually employ microwave irradiation and hydrothermal/solvothermal treatment [12,15,20]. In this review, because of their widespread use, bottom-up approaches will be explored more thoroughly, namely the hydrothermal/solvothermal and microwave-assisted routes of synthesis. A brief examination of other CQDs synthesis methodologies reveal approaches based on electrochemistry, sonochemistry, and laser ablation. Additionally, a brief overview of the recent biomass-based synthesis will be advanced. It should be noted that both the chemical and physical properties of the resulting CQDs are deeply dependent on the selected precursors and synthesis methodologies employed, the most affected being quantum yield (QY), oxygen/nitrogen percentage, size, crystallinity, and colloidal stability [6]. The main advantages and disadvantages for each method are illustrated in Table 2.

### 2.1. Hydrothermal/Solvothermal Synthesis

The hydrothermal/solvothermal synthesis is one of the most utilized approaches, since it allows the production of large quantities of CQDs with high QY and has a simple experimental arrangement [21]. This approach allows the usage of a broad temperature range that spans from room temperature to very elevated temperatures. Additionally, the hydrothermal/solvothermal synthesis enables the obtention of nanomaterials using experimental settings that employ high vapor pressures with a good production yield. Both the morphology and composition of nanosystems can be controlled by the manipulation of the vapor pressure of the reaction and by liquid phase or multiphase reactions, respectively [29]. Generally, small organic precursors are either dissolved or dispersed (in the case of polymers) in water, and then the obtained mixture is transferred to a Teflon-lined stainless-steel autoclave. Afterward, the exposure of the mixture to high temperatures will allow the formation of carbon cores that will constitute the foundation for the growth into CQDs [21].

**Figure 2:**
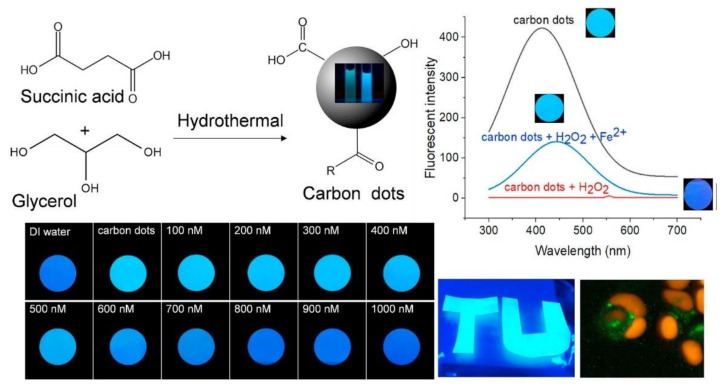
Representation of CQD hydrothermal synthesis, CQDs under UV illumination and practical application. Reprinted from ref. [33], with permission from Elsevier.

In 2019, Xue et al. [30] engineered lignin hybridized CQDs (L-CQDs) through a facile one-pot hydrothermal method using alkali lignin in the presence of different molar ratios of citric acid and ethanediamine and concluded that L-CQDs synthesized with equal molar ratio of citric acid and ethanediamine presented the highest fluorescence intensity. The L-CQDs displayed good water dispersibility and a near spherical morphology inferior to 10 nm in diameter, arranged mainly in interunit linkages and aromatic ring structures. As for the optical properties, the L-CQDs manifested an excitation dependent emission behavior, with emission maximum varying from 454 to 535 nm under excitation at 375–460 nm. An increase in excitation wavelengths resulted in red-shift in the maximum emission of the L-CQDs to a longer wavelength with a decrease in fluorescence intensity. The L-CQDs had good cellular internalization and displayed low cytotoxicity to HeLa cells. Synthesis of CQDs, utilizing aconitic acid (AA) as the carbon precursor and ethylenediamine as the co-doping reagent through a hydrothermal reaction, was carried out by Qian et al. [31]. The produced CQDs were water soluble and exhibited an excitation independent emission behavior. They presented a bright blue fluorescence and an absolute QY of 56.5% in aqueous solution. Furthermore, the CQDs intrinsic fluorescence was quenched by folic acid (FA) through their conjugated interaction, which lead to the development of a folic acid fluorescent sensor with a detection limit of 40 nm. Thus, CQDs were used in the detection of folic acid in pharmaceutical and food products with average recoveries in the range of 95.0–105.3% and relative standard deviation of less than 6.5%. Additionally, considering the qualitative differences in folic acid receptor expression on the surface of different cell types, the conjugated interaction of folic acid and CQDs allowed the distinction of Hela, SMMC-7721, and A549 cells. Therefore, the prepared FA-AA-CQDs could enter the cells by receptor-mediated endocytosis. Zhang et al. [32] reported the synthesis of chiral CQDs from cysteine, and citric acid through the hydrothermal method. The thus synthesized CQDs were constituted by 52.27% carbon, 14.45% nitrogen, 27.57% oxygen, and 5.71% sulfur. Moreover, the chiral CQDs were stable under several pH values, temperatures, and ionic strengths, and the location and intensity of the CQDs signal remained unchanged, verified by circular dichroism spectroscopy. Taking into consideration the chiral CQDs properties, they were utilized for the first time for the determination of the systemic effect of these nanomaterials on plants. Therefore, while it was determined that both D-CQDs and L-CQDs could promote the growth and photosynthetic capacity of the mung bean plant, D-CQDs manifested outstanding efficiency. Prathumsuwan et al. [33] produced multi-colored CQDs from succinic acid and glycerol through a one-step hydrothermal synthesis and without requiring acid or base catalysts. By varying the reaction time, blue and green-fluorescent CQDs could be obtained, with QY values of 11% and 7%, respectively. The blue-fluorescent carbon dots displayed turn-off fluorescence upon addition of H_2_O_2_ and turn-on fluorescence upon addition of Fe^2+^, and thus were evaluated as dual-mode sensors for the detection of these two substances. Therefore, CQDs were incorporated in a paper-based sensor system that determined Fe^2+^ and H_2_O_2_ concentrations as low as 30 μm and 400 nm, correspondently, in real water samples. The CQDs were also included in polymeric materials and tested for cell imaging applications. The schematic representation of the synthesis process and application is illustrated in Figure 2.

In 2020, Khan et al. [34] manufactured nitrogen and sulfur co-doped CQDs (NS-CQDs) in a facile one step hydrothermal synthesis using L-Lysine and thiourea. The resultant NS-CQDs were water soluble and exhibited a QY of 53.19%, with a strong blue fluorescence under excitation of a 365 nm wavelength light source. The NS-CQDs had an excitation dependent behavior and an average diameter size of 6.86 nm. The NS-CQDs were employed as a fluorescent probe relying on the Förster Resonance Energy Transfer (FRET) mechanism for the detection of picric acid in aqueous solution, revealing an adequate linear response to picric acid in the concentration range 1–10 μm with a detection limit of 0.24 μm. The concentration of picric acid in real water samples was also determined. Baragau et al. [22] reported a continuous hydrothermal flow synthesis of nitrogen doped CQDs (NCQDss) using citric acid and ammonia as precursors. Readily dispersed in water, the as-synthesized NCQDss displayed an excitation independent behavior with highest emission intensity at 441 nm, and also displayed a narrow Full Width at Half Maximum (FWHM). The NCQDss had an average size of 3.3 ± 0.7 nm. Due to the NCQDss substantial selectivity and sensitivity as a fluorescence sensor for chromium (VI) ions that was caused by the inner filter effect, the NCQDss were applied to the chemical sensing of Cr (VI) ions in aqueous solutions.

### 2.2. Microwave-Assisted Synthesis

Microwave-assisted technique is recognized as a quick, clean, and low-cost synthesis method that is a sustainable method identified with the Green Chemistry demands. It has been extensively utilized in the production of carbon-based nanomaterials, since the usage of microwave radiation allows an uniform heating and fast heating rates and interacts strongly with carbon materials; another feature that exalts the employment of microwave-assisted methods is the manipulation of internal and volumetric heating of materials [23]. Microwave-assisted techniques rely on causing heating at the molecular-level due to the alternating electric and magnetic fields that interact with the dipole moment of polar molecules present in a solvent. The heat that is generated through this interaction is termed dielectric heating and is absorbed by the carbon precursors. In opposition to conventional heating processes, microwave radiation infiltrates the target materials and generates thermal energy at atomic/molecular degrees, which in turn promotes steady and consistent volumetric heating [35]. Due to the ability of producing carbon-based nanomaterials in short-reaction times, low energy consumption, and accurate control of the reaction temperature, microwave assisted techniques have been extensively employed in green synthesis approaches [2]. However, despite the numerous advantages that can be enumerated for the use of microwave radiation in CQDs production, this method still presents some limitations. For example, the use of bulk metallic materials is restricted, since it causes interferences with the electromagnetic field to the point of making this kind of materials unusable either for synthesis or processing relying on microwave irradiation. However, this limitation is easily cleared by the usage of metal particles if the particle size is small enough to avoid the reflection of the incident waves [35].

**Figure 3:**
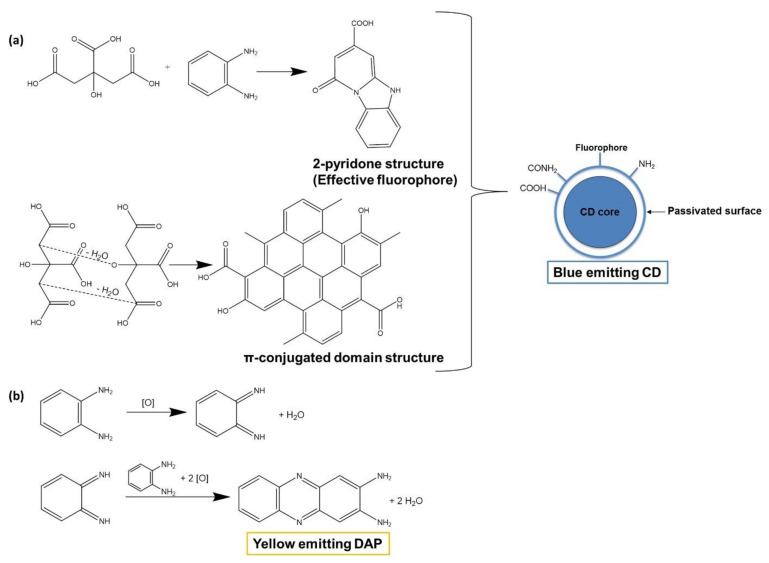
(**a**) Formation of CQDs from citric acid and *o*-phenylenediamine; (**b**) formation of DAP by oxidative dimerization of *o*-phenylenediamine. Reprinted from ref. [38], with permission from Elsevier.

Uriarte et al. [36] have synthesized CQDs through a one-pot microwave assisted synthesis using glycerol and urea. The synthesis was fast, having only lasted 4 min. The obtained CQDs presented an average diameter of 13.2 nm with about 9.8% of QY. CQDs also displayed significative photostability, since their fluorescent intensity did not suffer alterations after 1 h of continuous light irradiation at 345 nm, although CQDs fluorescence did manifest a pH-dependent behavior, exhibiting an optimal luminescence emission when the pH was lower than 4.0. The CQDs interaction with tetracyclines compounds was then evaluated, revealing a fluorescence quenching on the native fluorescence of the CQDs, which can be attributed to a static quenching, possibly correlated with the formation of a ground-state complex between tetracyclines and CQDs. Therefore, a fluorescence assay was developed for the quantification of tetracyclines, which showed good linearity range between 0.5 and 25 μm and a detection limit of 165 nm. The method was used for the analysis of urine samples with good recoveries (94.7–103%) and precision (4.6 RSD%). In 2019, Lu et al. [37] produced N-doped CQDs from gallic acid, citric acid, and ethylenediamine by a microwave assisted-synthesis. The synthesized CQDs fluorescence intensity was stable at different ion concentrations, but the prolonged exposure to ultraviolet light lead to a decrease in fluorescence. The CQDs maintained their fluorescence in the pH range between 5–9, and the maximum fluorescence intensity was observed when pH was 5. The QY of the developed CQDs was determined to be 25%. After testing for antitumor activity by MTT assays and in vivo antitumor experiments, it was revealed that CQDs retained the antitumor activity of their precursor, gallic acid. The CQDs presented an outstanding antitumor activity towards HeLa cells in a dose dependent manner. Hinterberger et al. [38] developed white light emitting CQDs by microwave-assisted synthesis utilizing as precursors o-phenylene-diamine and citric acid. The microwave irradiation originated CQDs with blue fluorescence and, also, the yellow-emitting molecule 2,3-diaminophenazine (DAP). A suggested molecular mechanism for the formation of CQDs and DAP is displayed in Figure 3. Hence, the resulting solution exhibits two fluorescence emission peaks at 430 and 560 nm, which correspond to CQDs and DAP, respectively. The size of the CQDs was determined by AFM to be 1.1 ± 0.3 nm. The emission of white light was achieved by varying the pH value of the solution, since the intensity ratio of both fluorescence peaks depends on pH, which is caused by the protonation state of DAP. At a pH value of 5.4, both blue and yellow emission intensities are similar and thus the light emitted is white.

Seifikar et al. [39] manufactured CQDs utilizing glucose and polyethylene glycol (PEG) by microwave-assisted synthesis. The reaction mixture was irradiated twice, first at 700 W for 15 min, and later after the addiction of 5 mL of NaCl 3.0 mol L^−1^, at 540 W for 2 min. The NaCl was used, since it triggers the precipitation of the dispersed CQDs. Characterization revealed the obtained product was comprised of both nano and micro-size particles. After the characterization, the CQDs were applied and tested in the adsorption of two cationic dyes, methyl violet (MV) and cationic blue (CB), present in water, revealing that the adsorption rate onto the obtained nanomaterial was swift, taking only a few seconds. The adsorption rate was found to be influenced by temperature. In 2020, Li et al. [40] synthesized far-red CQDs (FR-CQDs) by treating glutathione dissolved in formamide with ultrasounds and then submitting the mixture to microwave irradiation for 3 min at 800 W. The obtained FR-CQDs had a QY of 18.50%. After purification (centrifugation at 10,000× *g* rpm for 5 min, followed by dialysis (molecular weight cutoff of 3500 Da)), the FR-CQDs were mixed with a suspension of chloroplasts in a sucrose buffer solution for 30 min at 4 °C to obtain FR-CQDs/chloroplast complexes. FR-CQDs allow for a more efficient photosynthesis, since they mediate the conversion of ultraviolet A (UV-A) light into 625–800 nm far-red emission, which can be directly absorbed and utilized by chloroplasts. The prepared FR-CDs/chloroplast complexes were then tested in vitro and in vivo for the improvement of photosynthetic activity of the Roman lettuce. The in vitro model displayed higher electron transfer efficiency between PS II (photosystem II) to PS I (photosystem I), and the in vivo experiment revealed an increase of fresh and dry weights, which confirms higher electron transfer rate compared with the control group. In addition, red carbon dots (R-CDs) and blue carbon dots (B-CDs) were also synthesized. In 2018, Li et al. [24] have manufactured N- and S-co-doped carbon quantum dots (NSCQDss) via a single step microwave-assisted synthesis, using ammonium citrate and L-cysteine as precursors. Both reagents were firstly dissolved in 10 mL ultrapure water, followed by a 2-min sonication, allowing the formation of a transparent solution. The synthesis required a 750 W microwave oven and was completed in 2.5 min. The obtained NSCQDss exhibited a QY value of 64% and displayed bright blue fluorescence when exposed to an excitation wavelength of 353 nm, with an emission maximum at 426 nm. The NSCQDss presented an average size of 2 nm. Afterward, the synthesized NSCQDss were used for the analysis of levofloxacin in real water samples, with significative selectivity and sensitivity. Recoveries of levofloxacin in the real water samples were 98.6–106.8%, with a linear range of 0.01–70 mg L^−1^ and a detection limit of 5.1 μg L^−1^.

### 2.3. Others

#### 2.3.1. Electrochemical Synthesis

Generally, the electrochemical synthesis entails the use of three electrodes, the working electrode comprised by a carbon precursor, as well as the counter and reference electrodes. Different results may be obtained depending on the employed carbon precursor and the utilized experimental setup [12]. Concerning the most usual carbon precursors, carbon fiber, graphene and graphite are extensively used, making this synthesis approach of low cost and appliable to mass production of CQDs. This methodology also presents the advantage of utilizing common raw materials and not resorting to environmentally aggressive chemicals. Despite these advantages, the fact that the purification processes of the obtained CQDs are laborious and time-consuming is perceived as a significant drawback to this synthesis method [41].

Niu et al. [42] reported the synthesis of green-fluorescent N-doped carbon quantum dots (N-CQDs) prepared by a bottom-up electrochemical (EC) method, using pyrocatechol and ethylenediamine as both precursors and electrolytes. The QY value for N-CQDs was verified to be 30.6%. Then, the produced N-CQDs were employed in a turn ON–OFF–ON–OFF fluorescence assay for the monitoring of alkaline phosphatase activity, based on the specific competitive interaction of N-CQDs with Fe^3+^ and pyrophosphate anions, as well as the hydrolysis of pyrophosphate anions in the presence alkaline phosphatase. Huang et al. [43] developed ionic liquid-functionalized carbon dots (IL-CDs) using the ionic liquid 1-butyl-3-methylimidazolium tetrafluoroborate, via electrochemical method. These IL-CDs were then incorporated in a ratiometric fluorescent assay, which also included 2,3-diaminophenazine (DAP), for the evaluation of alkaline phosphatase activity based on FRET. IL-CDs presented a blue fluorescence signal at 470 nm and 2,3-diaminophenazine (DAP) had a yellow fluorescence signal at 570 nm.

#### 2.3.2. Sonochemical Synthesis/Ultrasonic Treatment

The sonochemical method is yet another green synthesis methodology, since it allows the production of large quantities of CQDs without the usage of harmful or toxic substances. The method consists in providing high-power ultrasonic waves to the reaction mixture, which in turn will cause cavitation, the formation of high-pressure vapor bubbles in the solvent [25]. The conversion of ultrasonic waves into mechanical energy allows the formation of CQDs through nucleating events, polymerization, and/or aromatization [15]. The regulation of the frequency and power of the ultrasonic waves, sonication time, and carbon precursors utilized is important to define the physical and chemical properties of the generated CQDs. Advantages to this technique are the single step synthesis of considerable amounts of CQDs and the fact that the sonochemical method is the only method for doping CQDs with bulk metals (including Ga, In, Bi, Sn, Pb, Cd, Sb, and Zn) [44].

In 2019, He et al. [45] have manufactured for the first time CQDs-based lubricants from citric acid, urea, and poly(ethylene glycol) (PEG), by sonochemical synthesis. The reaction mixture was submitted to ultrasonic treatment for 60 min at room temperature, producing CQDs with an average size of 2.38 nm and highly efficient lubricating properties. Lu et al. [46] reported the synthesis of CQDs via sonochemistry utilizing dopamine dissolved in dimethylformamide. The as-produced CQDs had a QY of 3.6% and a maximum emission at 434 nm upon 350 nm excitation. Size distribution ranged from 2.5–5.5 nm. The CQDs were used in the detection of Fe^2+^ ions both in water and in the interior of cancer cells, and also interestingly were shown to be temperature-dependent, allowing them to act as temperature sensors, both in water and in cells. Leong et al. [26] produced CQDs from curcumin dissolved in ethanol, resorting to the combination of mechanical and ultrasonic milling techniques. The average size of CQDs was 13.7 nm. The CQDs were then evaluated in vitro on their antibacterial activity, revealing a broad-spectrum bacteriostatic activity.

#### 2.3.3. Laser Ablation

Laser ablation has been used to synthesize nanoparticles since the nineties. It consists of solid targets immersed in either liquid (laser ablation of solids in liquids—LASL) or vapor. LASL presents the advantage of allowing the production of CQDs of substantial quality in a rapid process, with or without employing surface functionalization. By modifying experimental physical conditions, such as pulse duration, wavelength, repetition rate, and fluence of the incident laser or, on the other hand, the nature of the liquid media and target materials utilized, LASL allows the manufacturing of CQDs with distinct optical and chemical properties. Nevertheless, the deficiency in data correlating physical parameters that modulate aspects such as size or optical properties of the synthetized CQDs restricts laser ablation use in their synthesis. Additionally, despite identical experimental settings, reproducibility is often lower than expected, presenting a major challenge for laser ablation-based synthesis of CQDs [27,47].

Isnaeni et al. [48] fabricated toluene-soluble CQDs from coconut fiber resorting to laser ablation. Depending on the excitation wavelengths, the CQDs presented distinctive emission peaks at the wavelengths of 300, 440, and 540 nm, corresponding to the π orbital state, σ orbital state, and surface state, respectively. The transfer of energy between the surface state and the σ orbital state was considered very likely, while no correlation was found between π orbital state and the surface state. The electron decay time in the CQDs surface state was found to be very brief. Nguyen et al. [28] synthesized ultrasmall CQDs via double-pulse femtosecond laser ablation in solution, using graphite powders dispersed into ethanol. The minimum size of CQDs was ∼1 nm when the laser pulses approached electron-ion relaxation time, obtaining smaller CQDs than those produced via single-pulse ablation with the same laser fluence.

#### 2.3.4. Natural Sources/Biomass/Waste Products

The use of natural sources and waste products in CQDs synthesis qualifies underused and undervalued inexpensive resources to be converted in nanomaterials in a simple and cost-effective way. Several methods may be exploited to manufacture CQDs with properties tailored to the application in mind, such as microwave-assisted, electrochemical, confined pyrolysis, or solution chemistry methods. Those methodologies have the advantage of preventing CQDs agglomeration, as well as allowing the production of highly biocompatible CQDs. However, the usage of these kinds of starting materials presents the disadvantage of the necessity of surface passivation and doping methods to obtain higher QY. Additionally, it should be pointed out that the possibility of contamination from environmental pollution cannot be disregarded, particularly when the synthesized CQDs are to be used in biomedical applications. This problem can be overcome, once again, by surface passivation, providing, for example, a polymer layer over the CQDs [16,49].

Ren et al. [50] developed N-doped micropore CQDs passivated with formamide utilizing the pulsed laser ablation (PLA) method and using waste *Platanus* biomass as the carbon precursor. The N-doped micropore CQDs displayed dual-wavelength coexisting fluorescence emissions in the indigo-blue wavelength region, suggesting the transition of the excited electrons from the intrinsic π * orbital to the surface state produced from the saturation passivation. Characterization studies determined QY to be as high as 32.4% and fluorescence lifetime to be 6.56 ns, highlighting the importance of the nitrogen-containing functional group in the augmentation of the QY, when compared to CQDs without nitrogen in their composition. The produced N-doped micropore CQDs had an excitation independent emission behavior in various conditions such as varying pH values, temperatures, excitation wavelengths, salt ionic concentrations, and irradiation times. Posteriorly, the N-doped micropore CQDs were employed in cell bioimaging of HeLa cells, L02 cells, and macrophage cells and have been shown to be well internalized. In 2018, Devi et al. [51] reported the synthesis of CQDs from aloe-vera extract by a facile one-step pyrolysis method, with optimal synthesis conditions fixed at 190 °C and 20 min of synthesis time. The obtained product was subjected to dilution with water followed by the purification against water in a 0.3 kDa dialysis membrane. Characterization revealed the existence of carboxyl and hydroxyl functional groups on the CQDs surface, a bright blue luminescence under UV light and a QY of 12.3%. Additionally, the CQDs presented an excitation independent emission behavior. Following, the CQDs were applied for Fe (III) sensing in water and tested for their light-activated antibacterial activity against *Escherichia coli* and *Staphylococcus aureus*. Bhamore et al. [52] produced multi-color CQDs (blue, green, and yellow) using *Manilkara zapota* fruits, H_2_SO_4_, or H_3_PO_4_ at either 80 or 100 °C, and selected time settings that varied between 15 to 60 min. The synthesized CQDs had average sizes of 1.9 ± 0.3, 2.9 ± 0.7, and 4.5 ± 1.25 nm for blue, green, and yellow CQDs, respectively. The obtained CQDs demonstrated good dispersibility, presented good QY values (5.7%, 7.9%, and 5.2% for blue-, green-, and yellow- C-dots), and manifested excitation-dependent emission behavior. The CQDs were then used as imaging agents for observation of *Escherichia coli*, *Aspergillus aculeatus,* and *Fomitopsis* sp. organisms.

## 3. Structure Modifications

### 3.1. Heteroatom Doping

**Figure 4:**
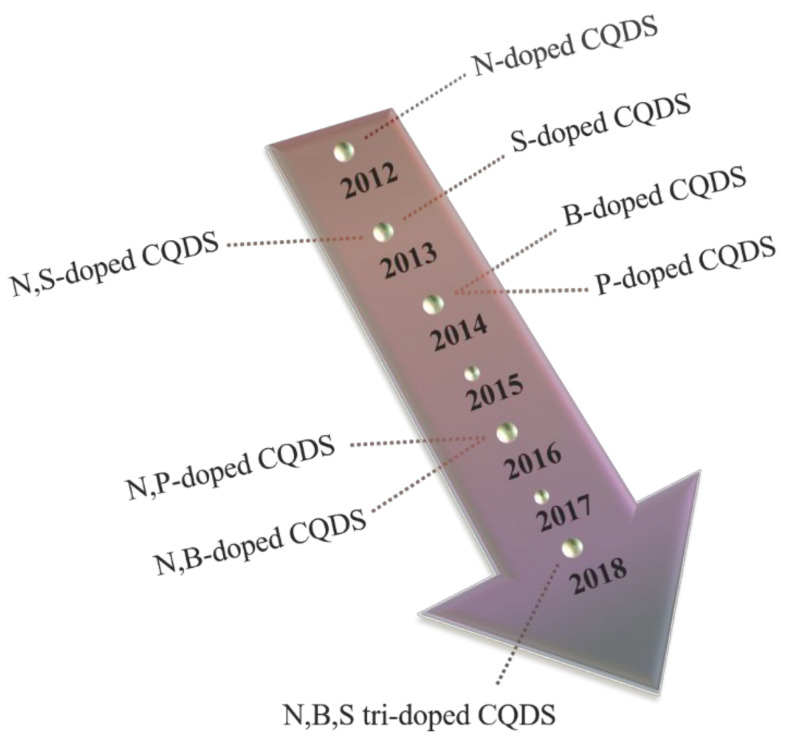
Heteroatom doping progress timeline.

The inclusion of both non-metallic and metallic elements in the CQDs is responsible for a shift in the electronic distribution, affecting the energy gap between HOMO (Highest Occupied Molecular Orbital) and LUMO (Lowest Unoccupied Molecular Orbital), and causing modifications on the surface configuration. Thus, the modulation triggered by the dopants is directly accountable for the properties exhibited by the synthesized CQDs and so, by manipulating both the nature and quantities of the elements employed, it is possible to boost and tune the fluorescence properties of CQDs. In terms of the type of dopant utilized, it is known that while non-metallic elements can reorganize the band arrangement of CQDs, metallic dopants are used to avoid unwarranted depletion of carboxyl and amino functional groups in the starting materials, particularly during the carbonization and dehydration steps of the synthesis, due to the chelation between the mentioned chemical groups and metal ions [53]. Considering that most metals present varying degrees of toxicity and are, therefore, inadequate for most biomedical and pharmaceutical applications, this section will provide a more in-depth vision of non-metallic heteroatom doping of CQDs. Recent progress in heteroatom doping has been illustrated in Figure 4.

#### 3.1.1. Single Heteroatom Doping

##### N-Doped CQDs

Considering the frequently modest QYs exhibited by “naked” CQDs and the fact that nitrogen atoms possess five valence electrons and similar atomic size to carbon atoms, N-doping is an approach usually utilized, since it bestows and/or enhances several of CQDs already exceptional properties [54,55].

Zhao et al. [56] have produced nitrogen-doped carbon quantum dots (N-CQDs) utilizing polyacrylamide, as it is a carbon- and nitrogen-rich precursor, by hydrothermal synthesis. Besides excellent water solubility, the N-CQDs possessed a QY of 23.1% and several surface functional groups, such as carboxyl acids and amines. Thus, the N-CQDs were evaluated as fluorescent nano-probes for sensitive and selective detection of dopamine. Through the conversion of dopamine into dopamine–quinone under alkaline conditions, a relationship between fluorescence quenching and the concentration of dopamine was established. A good linear correspondence was defined between these two parameters, and the concentration of dopamine in the range 0.1–200 μm was attained with a low detection limit of 0.05 μm. The developed method was then applied to the quantification of dopamine in urine samples. In 2020, Meng et al. [57] fabricated N-doped CQDs (N-CQDs) by one-pot hydrothermal synthesis from *p*-phenylenediamine and ammonia. The synthesized N-CQDs were uniform and monodispersed, with an average size of 3.2 nm and a QY of 13.2%. The CQDs manifested excitation dependent emission behavior. Then, the CQDs were used in a multi-sensing platform for the detection of chromium (VI), 2,4,6-trinitrophenol, and ascorbic acid, based on the inner filter effect (IFE). Bandi et al. [58] developed hydrothermally synthesized N-CQDs from *o*-phenylenediamine (OPD) and 2,5 pyridinedicarboxylicacid (2,5 PDC). The N-CQDs displayed a high QY value of 47% and excitation tunable emission behavior dependent on the pH value of the solution, which is caused by the participation of surface states, supporting their influence in the origin of excitation dependent nature. Furthermore, the as-prepared N-CQDs were employed in the individual determination of both Cu (II) and Fe (III) ions in spiked water, serum, and urine samples, by UV-visible spectra and fluorescence lifetime analysis.

##### B-Doped CQDs

Heteroatom doping of CQDs with boron atoms is accountable for a marked improvement in the fluorescence properties of CQDs [59]. Zhang et al. [60] have reported the synthesis of B-doped CQDs by the hydrothermal method using citric acid and boric acid as carbon and boron sources, respectively. The B-CQDs had an average size of 2.3 nm composed of multi lattice planes. Characterization studies revealed –COOH, –OH, C = O, and C-B functional groups and QY values of 30.85% and 17.92% for B-CQDs and plain CQDs, correspondingly. Afterward, the B-CQDs were tested as fluorescence probes for chemical control of amoxicillin in aqueous solution. Linear response between the enhancement of B-CQDs native fluorescence and amoxicillin concentration was observed when amoxicillin was present in solution in the range of 1.43–429.12 μmol L^−1^. Yong et al. [61] prepared boron-doped CQDs (B-CQDs) through a one-step hydrothermal method from phenylboronic acid. The as-synthesized B-CQDs presented an average size of 3.3 nm and a fluorescent excitation/emission profile at the wavelengths of 247/323 nm. The QY was determined to be 12%. Posteriorly, the B-CQDs were utilized as fluorescent probes for both sorbate and vitamin B12 determination in bread, vinegar or mineral water, and vitamin B12 tablets, vitamin drink, or mineral water, respectively. Other real samples were a source for interferences due to coexisting of food colorants.

#### 3.1.2. Co-Doping Multiplex Heteroatoms

##### N,P-co-CQDs

While single heteroatom doping with nitrogen grants access to active sites that can enhance optical properties of CQDs by creating surface defects due to carbon atoms replacement, some N-CQDs present low aqueous dispersity. On the other hand, the co-doping CQDs with nitrogen and phosphorous avoids alkaline, acidic, and metal ion interference, while favoring, at the same time, aqueous dispersity [62].

Liping et al. [63] prepared nitrogen and phosphorus co-doped CQDs (N,P-CQDs) from *o*-phosphorylethanolamine and citric acid via hydrothermal method. The N,P-CQDs displayed a QY of 8.17% and excitation dependent emission behavior, having an average size of 1.6 nm. Considering the N,P-CQDs fluorescence enhancement when in presence of Cadmium (II) ions, a fluorometric assay was developed for the quantification of Cd (II) ions, based on the chelation enhanced fluorescence that is induced by the formation of Cd(II)-N,P-CDs complex. Good linear response between Cd (II) concentration and the increase in N,P-CQDs fluorescence was observed when Cd (II) concentration ranged from 0.5 to 12.5 μm, and the method had a 0.16 μm detection limit. The detection of Cd (II) was performed in spiked serum and urine samples. Omer [64] reported the synthesis of nitrogen and phosphorus co-doped CQDs from glucose and polyethylenimine via a low-temperature carbonization route. The CQDs presented a 25% QY and a size distribution ranging from 2 to 9 nm. Additionally, the CQDs displayed excitation-independent fluorescence. Then, the CQDs were used as a fluorescent probe for the selective and sensitive detection of copper ions, based on the quenching of CQDs native fluorescence. The determined limit of detection was 1.5 nm. Liu et al. [65] developed nitrogen and phosphorus dual-doped CQDs (NP-CQDs) via a self-exothermic reaction using glucose, 1,2-ethylenediamine, and concentrated phosphoric acid as precursors. QY was determined to be 9.59%, and the NP-CQDs had an average size of 3.5 ± 0.2 nm. The as-produced N,P-CQDs presented excitation-dependent fluorescence behavior. Additionally, the N,P-CQDs were evaluated as fluorescent sensors for determination of curcumin in aqueous solution, obtaining a linear range of 0.5–20 µmol L^−1^ and a detection limit of 58 nmol L^−1^ (21.37 ng mL^−1^). The method was applied to drinking water and food samples. Additionally, the N,P-CQDs were utilized as fluorescent agents for cellular imaging and did not manifest noticeable cytotoxicity.

##### N,S-co-CQDs

Even though single heteroatom doping can be responsible for a modulation in the emission wavelengths of CQDs, such variation is not significant enough to reallocate their emission to longer wavelengths (superior to 600 nm). To attune the emission properties of CQDs, the insertion of N and S atoms can be employed, as such modification provides additional energy levels between π and π * of carbon atoms [66].

Liu et al. [67] manufactured N,S-doped CQDs using methionine and citric acid through hydrothermal synthesis. The as-synthesized CQDs manifested excellent water solubility due to the hydroxyl, carboxyl, and amino groups on the CQDs surface, and a QY of 13.8%. The CQDs had an average diameter of 5 nm and displayed an excitation dependent emission behavior. The average fluorescence lifetime of the CQDs was 3.67 ns. Additionally, the CQDs exhibited outstanding stability under different pH values and high ionic strength. CQDs were then employed in the bioimaging of Hep-2 cells. In 2018, Li et al. [68] produced N,S-doped chiral L (or D)-CQDs by hydrothermal treatment of L (or D)-cysteine. The CQDs had a QY value of 41.26% and displayed pH-dependent fluorescence intensity due to surface state involvement. The L-CQDs exhibited chirality-dependent enhancement in cellular glycolysis, despite being incapable of influencing the cellular ATP levels of T24 cells. Ding et al. [69] prepared N,S-CQDs via a one-step microwave method utilizing 1,6-hexanediamine dihydrochloride and dimethyl sulfoxide as precursors. The as-synthesized N,S-CQDs manifested a QY of 24% and excitation dependent emission behavior, emitting blue, green, yellow, and orange fluorescence as the excitation wavelength shifts to higher values. The average diameter of the N,S-CQDs was 4.35 nm. The N,S-CDs displayed very good thermal stability and under medium and acidic conditions suffered a fluorescence enhancement. Additionally, the N,S-CDs were employed in the detection of MnO_4_^−^ and Cr_2_O_7_^2−^ with detection limits of 0.34 and 0.23 μm, correspondingly.

##### N,B-co-CQDs

Co-doping CQDs with nitrogen and boron atoms enhances their fluorescence QY and seems to be responsible for the obtaining of CQDs with an excitation independent behavior [70].

In 2019, Jiang et al. [71] synthesized B,N-CQDs via hydrothermal synthesis using *p*-amino salicylic acid, boric acid, and ethylene glycol dimethacrylate as starting materials. The obtained B,N-CQDs had a QY of 19.6% and an average size of 5 nm. B,N-CQDs maximum excitation and emission wavelengths were 380 and 520 nm, respectively, and were shown to be highly stable under different pH values, ionic strengths, storage times, and prolonged exposure times to a 365 nm UV lamp. Afterward, the B,N-CQDs were used as fluorescent probes for the detection of the drug captopril, an angiotensin-converting enzyme (ACE) inhibitor used for the treatment of hypertension, based on an inner filter effect and a static quenching effect. The assay has been utilized for the detection of captopril in mouse plasma. Huang et al. [72] have manufactured N,B-CQDs via a modified hydrothermal method, utilizing 3-aminobenzeneboronic acid (APBA) and 1,2-ethylenediamine (EDA) as precursors. The resulting N,B-CQDs exhibited excitation/emission peaks at 400/510 nm and a fluorescence QY of 47%. Based on an inner filter effect on N,B-CQDs fluorescence, a fluorescence methodology for the detection of α-glucosidase activity and its inhibitors in water and living cells was established. Alizadeh et al. [73] have reported the production of nitrogen and boron-doped CQDs from phenylbronic acid, rhodamine B, and *o*-phenylenediamine (OPD) by hydrothermal synthesis. The CQDs presented a QY of 46% and an average size of 30 nm. The CQDs also exhibited excitation dependent fluorescence behavior. Under different pH values, the CQDs maintained constant fluorescence intensity. Furthermore, the CQDs were used as fluorescent probes for glucose determination in an RGB technique employing the RGB image capabilities of a smartphone.

##### Tri-Doped CQDs

To accomplish fine-tuning of CQDs optical properties, co-doping with three distinct elements can be applied as a versatile strategy to attain a more controllable adjustment of features such as excitation and emission wavelengths or QY values.

In 2020, Gao et al. [74] developed N/S/P co-doped CQDs through an ultrasonic-assisted synthesis, using thiamine pyrophosphate, a natural biological extract, as the sole donor of carbon, nitrogen, sulfur, and phosphorus atoms, in an alkaline solution and room temperature. The reaction was processed in 240 min. The synthesized N/S/P-CQDs were water-soluble, presented bright photoluminescence that was tunable by temperature, and displayed several functional groups, such as -OH, -COO-, -C-S, and -PO_3_. After the synthesis and purification/isolation by dialysis and freeze-drying, a 1 mg/mL aqueous dispersion of the N/S/P-CQDs was prepared. Moreover, the N/S/P-CQDs were tested as fluorescence probes for tetracycline detection based on the inner filter effect. The methodology showed high sensitivity for the detection of tetracycline in real samples, such as milk and tap water. Huang et al. [75] hydrothermally synthesized sulfur tri-doped CQDs (NPS-CQDs) resorting to *p*-aminobenzenesulfonic acid and tetrakis(hydroxymethyl)phosphonium chloride as precursors. Characterization studies determined the NPS-CQDs to be biocompatible when tested with HeLa MCF-7 cells during 48 h of incubation. The produced NPS-CQDs manifested excitation/emission maxima at 360 and 505 nm and exhibited good optical stability. Considering that chromium (VI) presents an absorption maximum at 350 nm, the inner filter effect on the blue fluorescence of the NPS-CQDs was used to produce a rapid, sensitive, and selective fluorometric assay for the determination of chromium (VI) in real waters samples and living cells. The detection limit was determined to be 0.23 μm, and linear response ranges from 1 to 500 μm chromate concentration range. Huang et al. [76] reported the synthesis of red emission nitrogen, boron, and sulfur co-doped CQDs (NBS-CQDs). The hydrothermal synthesis was processed by using 3-aminobenzeneboronic acid and 2,5-diaminobenzenesulfonic acid as precursor reagents. The NBS-CQDs exhibited a QY value of 11.6% and stable optical properties, even under varying pH values, ionic strengths, and long-time irradiation by UV light. It was also assessed that the synthesized NBS-CQDs presented an excitation independent emission behavior. Owing to the intense quenching on the fluorescence of NBS-CQDs caused by the presence of silver ions, NBS-CQDs were employed in the quantification of Ag^+^ ions in a high sensitivity and selectivity fluorescence assay. The quenched fluorescence of NBS-CDs was recuperated after the interaction between Ag^+^ ions and *L*-Cysteine, thus creating an “ON–OFF–ON” fluorescent probe, which was used in the specific determination of *L*-Cysteine in human urine samples and human plasma samples. This way, low detection limits of 0.35 mmol L^−1^ for Ag^+^ ions and 0.045 mmol L^−1^ for *L*-Cysteine were obtained. Additionally, this principle was applied to visual detections of Ag^+^ ions and *L*-Cysteine in HeLa and MCF-7 cells.

### 3.2. Surface Functionalization

Considering that surface functional groups are responsible for numerous properties of CQDs, and that the interaction between those groups and other entities, either biological or not, is determinant for CQDs application, surface functionalization is mandatory to obtain high-quality CQDs. When functionalizing the surface, CQDs objective application must be considered, since features such as surface charge status will directly affect both the electrostatic interactions with the target and CQDs fluorescence properties. However, despite the advantages of surface functionalization, the process is often challenging. Several approaches have been entailed to engineer CQDs surface, but the most common consist of binding small organic or biological molecules to the CQDs surface either through covalent bonds, or non-covalent interactions, such as electrostatic interactions or hydrogen bonds. Regarding covalent bonds strategy, carbodiimide chemistry (EDC/NHS) is often employed, as it allows the conjugation of organic, inorganic, biological, or polymeric compounds, presenting either amine or carboxyl functional groups, onto the surface of CQDs, whose surface is coated with carboxyl functional groups. The usual protocol for this reaction consists of activation of carboxyl groups by ethyl(dimethylaminopropyl)carbodiimide (EDC)/N-hydroxysuccinimide (NHS), followed by reaction with amino groups [77]. Yet, despite the practicality of this reaction, the hydrolysis of both amine and imino-reactive o-acylisourea structure leads to the formation of products that are frequently unstable in water [78]. Another strategy applied in CQDs surface functionalization other than binding ligands to CQDs surface is passivation with polymers. Considering the importance of surface defects, and functional groups in fluorescence emission processes, it is expected that polymer passivation would lead to an enhancement of CQDs fluorescence properties and the obtainment of superior QY values. By protecting surface functional groups, polymer passivation also shields those groups from processes such as agglomeration or oxidation that could cause a decrease in QY, contributing to an augmentation of CQDs stability. Moreover, the passivation with polymers is responsible for the narrowing of emission bandwidths, significant increases in emission lifetimes, as well as the perfecting of other optoelectronic properties [79]. The grafting of CQDs onto synthetic and biocompatible polymers also prevents agglomeration, and since generally synthetic polymers do not hinder CQDs fluorescence, due to their transparency in the visible region of the electromagnetic spectrum, merging synthetic biocompatible polymers with CQDs creates a fluorescent nanomaterial with excellent biocompatibility and exquisite optical features [80].

#### 3.2.1. Functionalization with Small Organic Molecules

**Figure 5:**
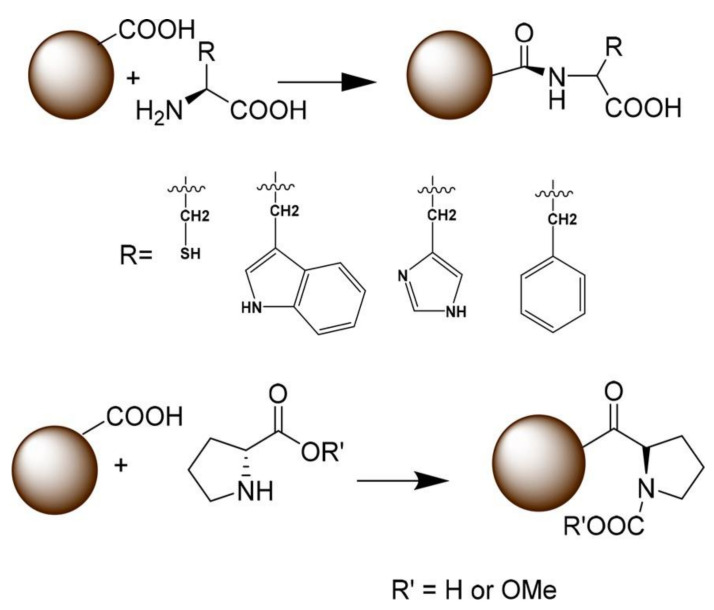
Schematic representation of the conjugation of amino acids on the CQDs surface. Reprinted with permission from ref. [82]. Copyright 2018 American Chemical Society.

In 2019, Abu Rabe et al. [81] synthesized three groups of CQDs by a reflux method. The first group comprised CQDs formed from either 2,2-(ethylenedioxy)bis(ethylamine) (EDA) or 3-ethoxypropylamine (EPA), to establish the effect of different terminal groups/charges on their photo-activated antibacterial activities. The second CQDs group was produced using polyethylenimine (PEI) or the mixture of citric acid and PEI, to determine the effects of CQDs surface charges vs. fluorescent QY on their antimicrobial activities. The third group included CQDs obtained from functionalization with PEI 1200 or PEI 600 and was used for the testing of the effect of molecular weight increase due to surface passivation on their antimicrobial activities. It was concluded that in the first group, CQDs obtained from 2,2-(ethylenedioxy)bis(ethylamine) were more efficient, which could be credited to the positive charges of the amino groups, favoring the interaction between CQDs and bacteria. Analysis of the second group revealed that the same surface charge effect was responsible for a better antibacterial performance, in detriment to QY values. Finally, in the third group, it was established that CQDs functionalized with PEI 600 displayed a more vigorous antibacterial activity. Ostadhossein et al. [82] obtained CQDs from sugars via microwave-assisted synthesis. Then, chiral capping was achieved by carbodiimide-mediated activation of the carboxylates on the CQDs surface, utilizing N-hydroxysuccinimide (NHS) and 1-ethyl-3-(3(dimethylamino)propyl) carbodiimide (EDC). Subsequently, covalent modification using cyclic α-amino acids was performed, resorting to proline (Pro), phenylalanine (Phe), histidine (His), tryptophan (Trp), alanine (Ala), and proline methyl ester (ProOMe). A schematic representation of the conjugation of amino acids on the CQDs surface is illustrated in Figure 5. This operation resulted in chirality inversion, which was confirmed by circular dichroism studies. This inversion could be generated by the production of a strained intermediate during surface conjugation of amino acid via carbodiimide coupling.

Kim et al. [83] have prepared soluble CQDs by one-pot hydrothermal synthesis, using L-lysine and L-glutathione. The CQDs showed excitation dependent emission behavior and stability at high temperatures (90 °C), as well as over prolonged periods (over 90 days). The CQDs fluorescence quenching caused by the formation of a complex between thiophenolate anions present on the CQDs surface and metal ions was employed in the selective detection of lead ions in human serum. The quenching effect was generated after the addition of lead ions due to Pearson’s hard and soft acid–base principle (HSAB), leading to the transfer of an electron from the conductance band (CB) of CQDs to lead ions. In 2019, Rossini et al. [84] manufactured CQDs resorting to citric acid and tyramine by a one-step microwave-assisted method. The resulting CQDs had an excitation independent emission behavior, suggesting the photoluminescence was originated from the quantum confinement effect. The incorporation yield for tyramine was calculated to be 74%, leaving the unreacted tyramine free to interact with CQDs, causing fluorescence quenching, or it could react with other free tyramine, leading to a non-radiative reaction. The tyramine functionalized CQDs were implemented in paper-based support, and used to monitor glucose in biological samples, by exploiting the H_2_O_2_ formed upon enzymatic reaction with glucose oxidase.

#### 3.2.2. Functionalization with Biological Molecules

In 2020, Fahmi et al. [85] synthesized hydrophobic CQDs by a pyrolysis process utilizing tartaric acid and L-tyrosine as starting materials. Afterward, human serum albumin (HSA) was mixed with CQDs and ultrasonicated to drive the CQDs into the hydrophobic core of the HSA. By providing harmonic pressing and expanding force to HSA, the ultrasonic waves facilitated the access of CQDs to the hydrophobic core of HSA when the macromolecule gets the expanding force. The CQDs were employed as staining agents for the HeLa cancer cell via both active and passive targeting. Sahu et al. [86] have fabricated CQDs with citric acid, urea, and boric acid via microwave-assisted synthesis. Furthermore, the as-prepared CQDs were submitted to surface modification with bovine serum albumin (BSA), promoted by continuous stirring for 24 h. Since BSA is a native fluorescent molecule, fluorescence bands at 444 nm and around 300 nm were observed, caused by CQDs and BSA modified CQDs, respectively. BSA fluorescence is mainly attributed to two tryptophan (Trp) residues situated on the protein surface. The BSA modified CQDs were utilized for the sensitive and selective detection of Pb (II) ions in pure solutions, in the range 1–10 ppb concentration (limit of detection of 0.08 ppb). Yang et al. [87] obtained CQDs using citric acid, folic acid, and branched polyethyleneimine (BPEI), which were dissolved in water by ultrasonic oscillation treatment. The mixture was then submitted to hydrothermal heating. For the covalent binding of β-cyclodextrin, functionalization of CQDs with 3-aminophenylboronic acid (3-APBA) was first executed via EDC/EHS coupling. Β-cyclodextrin (β-CD) were then covalently bond with APBA-CQDs, in an alkaline environment, by the formation of cyclic boronic esters between cis-diols of β-CD and boronic acid. Moreover, doxorubicin (DOX) was loaded into the previously prepared CQDs to create a multifunctional nanoplatform DOX-β-CD/CQDs for the controlled targeting, delivery, and release of the anti-cancer drug doxorubicin. Chen et al. [88] prepared nitrogen-doped CQDs (NCQDss) via an electrolytic method with graphite electrodes and ammonia aqueous solution as the electrolyte. After the synthesis, the N-CQDs were dispersed in water with the assistance of ultrasonication. Then, immobilization of horseradish peroxidase (HRP) was performed by adding N-CQDs to the solution of HRP in PBS at pH 7.0 and stirring (150 rpm) at 4 °C for 24 h. Adsorption, covalent attachment, and entrapment were the proposed mechanisms for the immobilization of HRP onto N-CQDs surface, through covalent bonding, hydrogen bonding, and hydrophobic interaction. The near-surface amino and carboxyl groups of HRP can effectively interact with the carboxyl, aldehyde, and amino groups of N-CQDs by hydrogen bonding or amido bonds, whereas hydrophobic groups of N-CQDs and the hydrophobic domain of HRP can promote a hydrophobic interaction that further aided HRP adsorption. The obtained N-CQDs@HRP were revealed to promote biomass and pigment contents of *Arabidopsis thaliana* seedlings under Pb, Cd, and saline stress conditions, proving the enhanced effect of HRP when functionalized with N-CQDs.

#### 3.2.3. Functionalization with Polymers

He et al. [89] have reported the synthesis of two cationic polymer-derived CQDs (Taea-CQDs and Cyclen-CQDs) by hydrothermal method. The polymers Pcyclen or Ptaea and citric acid were used as starting materials. Both Pcyclen and Ptaea had been previously prepared through an epoxide ring-opening polymerization, an effective synthetic approach that has the potential to boost high biological compatibility. The passivated CQDs emitted bright blue luminescence under UV light at 365 nm wavelength, in an excitation dependent emission manner. The CQDs presented a narrow FWHM, suggesting narrow size nanoparticles distribution. The average sizes of CQDs were measured as 1.8 ± 0.4 nm for Taea-CQDs and 5.4 ± 2 nm for Cyclen-CQDs. However, CQDs displayed a low QY value of 3.25% and 1.73% for Taea-CQDs and Cyclen-CQDs, respectively. They were then used as cell imaging agents for real-time detection of HeLa cell’s DNA transfection process. Janus et al. [90] manufactured poly(L-lysine)-based CQDs via a microwave-assisted synthesis. Firstly, the starting material poly(L-lysine) was prepared by the mixture of L-lysine and propylene carbonate, which was then submitted to microwave irradiation for 120 min at a temperature of 240 °C. Afterward, 35% H_2_SO_4_ was added to the previously prepared mixture and once again submitted to microwave irradiation. The synthesized CQDs exhibited a pH-dependent fluorescence emission, with a maximum QY inferior to 15%. The CQDs were utilized to monitor various biomolecules and metal ions. Free radical removal activity was also evaluated. In 2019, Arsalani et al. [91] fabricated polyethylene glycol (PEG) passivated fluorescent CQDs (CQDs-PEG) via microwave-assisted synthesis, utilizing gelatin and PEG as starting materials. CQDs-PEG presented a QY of 34% and an excitation dependent emission behavior. The average size of CQDs-PEG was estimated to be 6 nm. The as-prepared CQDs-PEG were afterward loaded with the drug methotrexate to develop an anticancer drug nanocarrier against MCF-7 cells. Li et al. [92] prepared polymer functionalized CQDs via the reflux method, using citric acid as a carbon source and Tris-HMA as a nitrogen source. After the CQDs synthesis and polymer preparation, the CQDs were functionalized with catechol-terminated hydrophilic poly(poly(ethylene glycol) methyl ether methacrylate (PPEGMA) or hydrophobic polystyrene (PS) by the mussel chemistry, obtaining CQDs@PEGMA and CQDs@PS, correspondently. The procedure relies on the principle that at room temperature, the mixing of reactants triggers the reaction while maintaining the inherent properties of the substrates. The Schiff base condensation/Michael addition reaction enhanced the optical and physical properties of bare CQDs through the polymer anchoring and PS that, being both hydrophobic, could grant hydrophobic features to otherwise hydrophilic CQDs, expanding their application range. The CQDs@PEGMA and CQDs@PS were later incorporated in a white LED.

## 4. Purification/Separation

Considering what has been mentioned previously about the influence that CQDs’ chemical and physical characteristics have on their properties and interactions’ potentialities, it is understandable that procedures that allow the purification or separation of different CQDs populations from the complex mixture that is formed during synthesis is one of the most important processes in CQDs production. However, although multiple synthesis methods have been detailed in the literature, the deficiency in purification methodologies is concerning. Among the methods described in the literature for the purification/separation of CQDs are dialysis, reversed-phase high performance liquid chromatography, electrophoresis, density gradient ultracentrifugation, pH-controlled cloud-point extraction technique, gel column size-exclusion chromatography, and others. The most utilized purification method for CQDs is, undoubtedly, dialysis. However, despite this, there are still no standards or guidelines either for the dialysis time or the molecular weight cut-off (MWCO) of the dialysis membrane, and other less utilized methods suffer from the same lack of information. Moreover, even though often purification methods are employed after CQDs synthesis, very few works particularize the conditions established for said purification. In the present paradigm of the CQDs research field, the attempt to utilize unpurified or inadequately purified CQDs to interpret reaction or formation mechanisms, and fluorescence emission generation mechanisms, leads to poor misinformation of data or warped data interpretation, with more questions raised than clarified.

### 4.1. Dialysis

Dialysis is the most used purification methodology employed. It relies on the unequal diffusion of small molecules or dissolved ions across the pores of semipermeable membranes, stemmed from the movement of substances from high concentration areas to low concentration areas, leading to separation.

In 2019, Chen et al. [93] have evaluated the appropriate time needed for efficient CQDs separation by dialysis resorting to HPLC. The CQDs were synthesized via a microwave-assisted method using citric acid as the sole precursor. The as-prepared CQDs displayed an excitation dependent emission behavior. Then, the decrease in the amount of residual synthesis by-products during the dialysis process was accompanied by HPLC. It was found that at least 120 h are required for the complete removal of the mentioned by-products from the CQDs solution. In fact, these monitoring studies allowed the authors to conclude about the very low yield of the synthesis that otherwise it would not be detected. Additionally, membranes with MWCOs inferior to 1 kDa are prone to trap the by-products, leading to an inefficient purification. Thus, it is was concluded that many reported studies in the literature did not comply with the referred specifications, either by employing dialysis membranes with too small MWCO or by not performing the dialysis procedure for an adequate amount of time. It was also found that solutions of CQDs derived only from citric acid contained at least three kinds of CQDs, demonstrating that even synthesis carried out solely with one carbon source can originate complex CQDs solutions. Moreover, the attempt to interpret the chemical composition or optical properties of CQDs present in different fractions of the HPLC chromatogram without a suitable dialysis process only leads to uncertain conclusions.

### 4.2. Reversed-Phase High Performance Liquid Chromatography

Reversed-phase high performance liquid chromatography (RP-HPLC) is the most common HPLC separation technique and is based on the hydrophobic interactions between sample molecules and the ligands on the chromatographic support. In RP-HPLC, the polarity of the mobile phase is superior to the one presented by the stationary phase. Additionally, HPLC carries the advantage of allowing the obtaining of large quantities of fractioned CQDs, promoting the efficient characterization and understanding of optical properties, as well as incentivizing their usage on more feature-exigent applications [94].

Liu et al. [95] have isolated CQDs by column chromatography and a binary gradient elution via RP-HPLC. The CQDs were synthesized from citric acid and urea via a calcination reaction. The isolation revealed that the obtained CQDs sample was composed of several CQDs fractions containing disparate proportions of carboxyl and amine functional groups, which in turn directly affects CQDs fluorescence properties. It was also established that by simply varying the precursors ratios, different polarity CQDs could be prepared.

### 4.3. Electrophoresis

Electrophoresis can be described as the movement that leads to the separation of charged particles under the effect of an electric field. The mobility of the particles is conditioned by parameters such as particle charge, size, and shape, as well as the temperature in which the electrophoresis is performed.

Kokorina et al. [96] performed a gel electrophoresis separation of fluorophores created during CQDs synthesis and studied possible origins for their light emission. Following the premise that quite often, the bright fluorescence displayed by some CQDs might be not be completely related to the particles themselves, but to molecular fluorescent species such as 1,2,3,5-tet rahydro-5-oxo-imidazo[1, 2-a]pyridine-7-carboxylic acid (IPCA), the effect of the precursor ratio on the optical properties of CQDs and in the formation of fluorescent species has been evaluated. Therefore, a hydrothermal synthesis was carried out using citric acid (CA) and ethylenediamine (EDA). Results showed that gel electrophoresis is successfully capable of separating fractions presenting excitation-dependent and independent emission behavior and also provided a correlation between both fluorophore QY and optical properties, and the selected experimental conditions, such as the precursor employed.

### 4.4. Density Gradient Ultracentrifugation

Density gradient ultracentrifugation is based on the density differences of the suspended particles and the gradient of the medium, which is formed by layering solutions of different densities. During centrifugation, the particles may either ascend or descend, settling in the layer with a corresponding density. Generally, long-term centrifugation is needed, and the density slope of gradient media in the centrifuge tube affects the resolution, as shallower centrifuge tubes allow for a better resolution [97].

### 4.5. Others

In 2020, Beiraghi et al. [98] purified and fractioned CQDs using a pH-controlled cloud-point extraction (CPE) technique. The CQDs were prepared using citric acid as the sole precursor via a hydrothermal synthesis. Firstly, CQDs were separated from their precursors by two steps. Initially, CQDs were transferred to centrifuge tubes and had their pH adjusted to values ranging from 1 to 12 using either HCl or NaOH. Later, Triton X-114 solution was added to the tubes that were then transferred to a thermostatic water bath at 40 °C, for 10 min. The process of the aqueous and surfactant-rich phase separation was speeded by centrifugation for 10 min at 3000 rpm. The second step involved the addition of water to surfactant-rich phases, after their separation through decantation, and pH adjustment to 7. Then, the CPE procedure was once again performed, and the supernatants obtained were collected. The fractioning of CQDs followed a similar experimental process and allowed the obtention of two fractions, namely, f1 and f4, in which the subscript refers to the pH values of aqueous phase. The fractionation was found to rely on the surface chemistry of the CQDs, a parameter that also has a significant effect on CQDs fluorescence emission. Uthirakumar et al. [99] developed a purification method alternative to dialysis, which allowed the isolation and recovery of over 80% of CQDs. The CQDs were synthesized by the reflux method using carbon black pigment dissolved in a mixture of H_2_SO_4_ and HNO_3_. The obtained CQDs had an average size of 2.6 nm. The isolation was carried out using a water-immiscible organic solvent, *n*-butanol. After the dilution of the previously prepared CQDs with distilled water, *n*-butanol was added. Both immiscible layers were stirred together for a controlled period and then separated using a separating funnel. Then, the top CQDs rich organic layer was collected, and the bottom layer was washed with *n*-butanol for two more times. Afterwards, the collected organic layers were mixed and swiftly washed with water for the removal of the free acid and salts. Kokorina el al. [100] reported the fractionation of a CQDs mixture by gel column size-exclusion chromatography. The CQDs were manufactured by the hydrothermal method using dextran sulfate sodium salt (DSS) as the starting material. Then, the as-prepared CQDs were submitted to gel column size-exclusion chromatography, resulting in 48 CQDs fractions (each of 0.7 ml) that displayed distinct optical properties. The 48 isolated CQDs fractions were comprised of at least three types of CQDs. The first kind of CQDs showed maximum emission at 410–420 nm, the second at 490 nm, and the third at 530 nm. Hinterberger et al. [101] have performed the purification of CQDs by column chromatography and have proposed a structural elucidation of the isolated CQDs. The CQDs were synthesized via hydrothermal method using citric acid (CA) and DL-cysteine, under different reaction conditions and reagent molar ratios. The purification of CQDs was achieved by employing a silica column with a water/acetonitrile (1:1) eluent. The isolated fractions were found to possess variable amounts of fluorescence emitting species, which was directly related to synthesis conditions employed, as well as the retention time. The obtained fractions contained free fluorophores 5-oxo-3,5-dihydro-2H-thiazolo [3,2-a] pyridine-3,7-dicarboxylic acid (TPDCA), fluorophores connected to CQDs surface and low-fluorescent carbon particles without connected fluorophores, and a higher number of free fluorophores was observed when the retention time was low. Moreover, harsher synthesis conditions appeared to lead to the destruction of free fluorophores, reducing the concentration of fluorescent CQDs. The highest QY value of 70% was obtained using mild experimental conditions.

## 5. Characterization Methods

**Figure 6:**
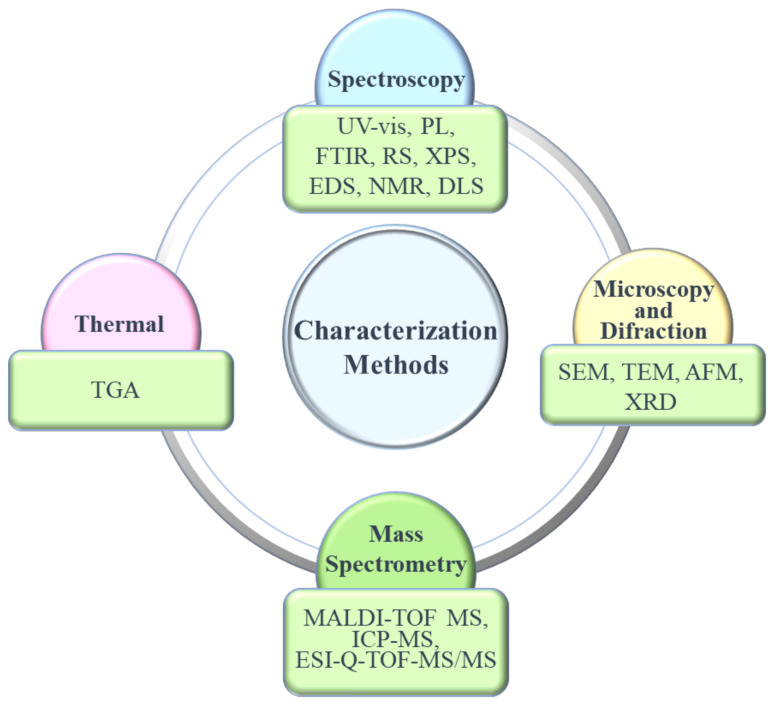
Diagram portraying common characterization methods of CQDs.

Just as important as purification/separation techniques, characterization methods are essential to assess CQDs’ features. Despite a better understanding of CQDs’ properties such as structure arrangement, morphology, chemical nature, and physical characteristics, much work remains to be done to completely elucidate CQDs’ characteristics as a nanomaterial. Part of the challenge of such endeavor is steamed from the fact that CQDs exist as a complex mixture that embodies several distinct elements with disparate sizes and surface functional groups [102]. In this section, some of the most used characterization methods (represented in Figure 6) will be examined in a brief and yet elucidative way, providing a much-needed overview of the various techniques employed in CQDs characterization. For this review, we will focus on microscopic and spectroscopic methods, since they are the most practical for routine characterization and provide a large amount of valuable information about CQDs features.

### 5.1. Microscopy/Diffraction

#### 5.1.1. Transmission and Scanning Electron Microscopies (TEM and SEM)

**Figure 7:**
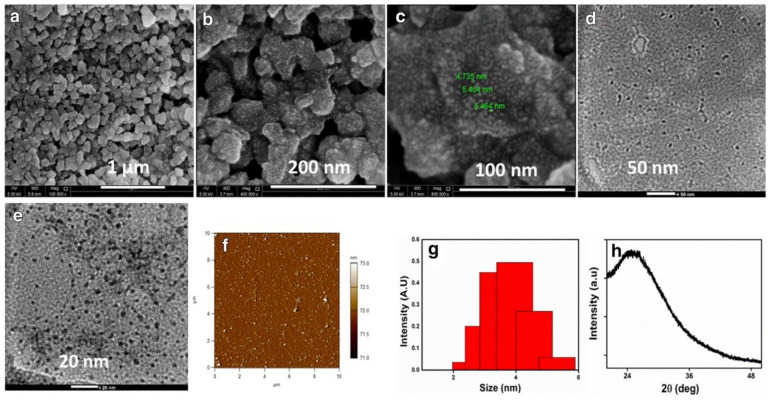
(**a**–**c**) SEM of CQDs; (**d**) and (**e**) TEM of CQDs; (**f**) CQDs size distribution; (**h**) XRD of CQDs. Reproduced with permission from ref. [105], published by Springer Open, 2020.

TEM relies on the incidence of a highly energetic electron beam upon a very thin sample to image and analyze the structure of nanomaterials with an atomic-scale resolution. Electromagnetic lenses are utilized to focus the electrons, and the image is attained either on a fluorescent screen or recorded on film or digital camera. TEM is one the most employed techniques in the characterization of carbon nanomaterials, as it allows the distinction of similar-looking nanomaterials that, otherwise, might be confused when resorting to scanning electron microscopy [103]. SEM uses focused electron beams, focusing incidental electrons onto the sample, producing electron scattering triggered by the interaction between the electrons and the sample atoms [104]. TEM and SEM share several features, and both are capable of discerning particle agglomeration to assess if adequate particle dispersion was accomplished. However, TEM is preferred in situations where the particle size exceeds the resolution of SEM, as TEM possesses higher resolution power. Figure 7a–e contains examples of SEM and TEM images of CQDs.

#### 5.1.2. Atomic Force Microscopy (AFM)

AFM works based on raster-scanning a cantilever-tip ensemble excited at a fixed frequency over the sample surface, probing the generated interaction force with piconewton sensitivity. The oscillation amplitude of the sharp tip ensemble is employed as a feedback parameter during the imaging of the sample topography [106,107]. In comparison to both SEM and TEM techniques that generate two-dimensional CQDs images, AFM also supplies three-dimensional images of the CQDs (Figure 7f), allowing the obtaining of morphological information, as well as the estimation of CQDs size by the random assessing the height of a fixed number of CQDs [108].

#### 5.1.3. X-ray Diffraction (XRD)

XRD is often used for the characterization of crystalline materials, and its working principle is that the interaction between X-rays and crystalline materials creates constructive interference that allows the identification of the materials based on their diffraction patterns. From the values of peak positions, areas, and FWHM, it is possible to calculate CQDs percentage crystallinity [109]. A representative example is depicted in Figure 7h.

### 5.2. Spectroscopy

#### 5.2.1. Ultraviolet-Visible (UV-vis) Spectroscopy

UV-vis spectroscopy of CQDs can be used to aid in the determination of CQDs surface functional groups, by clarifying interactions between these groups, particularly conjugation. For example, an absorption peak at 275 nm corresponds to n → π * transition associated to C = O bond, and a peak at 225 nm is caused by the π → π * transition related with aromatic sp^2^ domains [110].

#### 5.2.2. Photoluminescence (PL) Spectroscopy

**Figure 8:**
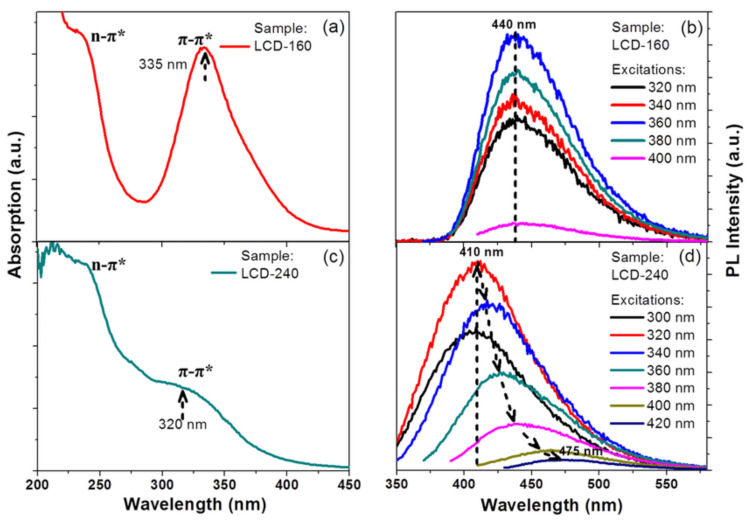
(**a**) and (**c**) UV-Vis absorption spectra of CQDs obtained at reaction temperature 60 and 240 °C; (**b**) and (**d**) PL spectra demonstrating independent and dependent behavior. Reproduced with permission from ref. [111], published by Scientific Reports, 2014.

The PL spectra of CQDs not only permit the assessment of the uniformity of CQDs chemical characteristics, as a large bandwidth emission is often related to mixed populations of CQDs, but also determine if the fluorescence emission is excitation wavelength dependent or not. Examples of excitation dependent or independent behavior can be observed in Figure 8. This characterization is very important as it allows for a better understanding of the possible fluorescence origin, as the intervention of surface states seems to generate excitation dependent emission behavior, and an independent behavior appears to be correlated with passivated surface states [7,111]. Additionally, the combination of information collected through UV-vis and PL spectroscopy can be used to assess the QY value of CQDs [112].

#### 5.2.3. Fourier-Transform Infrared Spectroscopy (FTIR)

**Figure 9:**
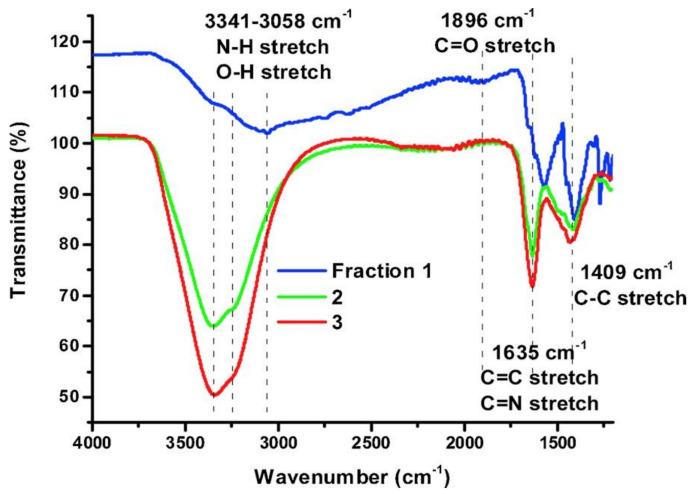
FTIR spectra of three CQDs fractions obtained from the same synthesis. Reprinted from ref. [114], with permission from Elsevier.

FTIR is one of the gold-standards in the determination of the functional groups, as it allows their distinction based on the vibration of interatomic bonds. Thus, absorption around the 3210–3640 cm^−1^ region can be ascribed to O-H, primary amines generate absorption around the 1580–1660 cm^−1^ region, and peaks at 1690–1760 cm^−1^ that are associated with absorption at 1080–1300 indicate the presence of carboxylic acid functional groups [113]. As hydroxyl, carboxyl, and amine functional groups tend to predominate in different proportions in CQDs surface, the usage of FTIR is an advantage in their characterization. In Figure 9 is depicted a FTIR spectra of previously fractioned CQDs, demonstrating that a single synthesis originates several CQDs populations.

#### 5.2.4. Raman Spectroscopy (RS)

Raman spectroscopy is a commonly employed as non-invasive and non-destructive method for CQDs characterization. The G band (graphitic band) is related to the E_2g_ mode of graphite and is associated with the vibration of sp^2^ carbon atoms in a two-dimensional hexagonal lattice, while the D band is generated by the vibrations of dangling bonds on carbon atoms in the termination plane of either disordered graphite or glassy carbon. By determining the ratio of the intensities of the disordered D band and crystalline G band (D/G), it is possible to evaluate the purity (degree of disorder or graphitization) of the studied nanomaterial [115].

#### 5.2.5. X-ray Photoelectron Spectroscopy (XPS)

XPS is a surface analysis technique particularly relevant as it provides information about the electronic state of the elements, elemental analysis of the outermost atomic layers, and surface features of a given nanomaterial. XPS relies on the ejection of photoelectrons of inner shells and the measurement of their kinetic energy, which is caused by the irradiation of the sample with X-ray photons. It is capable of detecting all elements except for hydrogen, and often does not present enough spatial resolution to study individual nanoparticles [116].

#### 5.2.6. Energy Dispersive Spectroscopy (EDS)

EDS is a useful method when the sample size is small and provides qualitative and quantitative information on the elemental composition of the examined material. After the surface atoms are excited by an electron beam, the emission of X-rays with characteristic wavelengths is analyzed by energy dispersive detectors that identify the atomic structure of the sample [117]. EDS can also be used to monitor CQDs purity, as impurities and unwanted elements can be easily detected.

## 6. Applications

**Figure 10:**
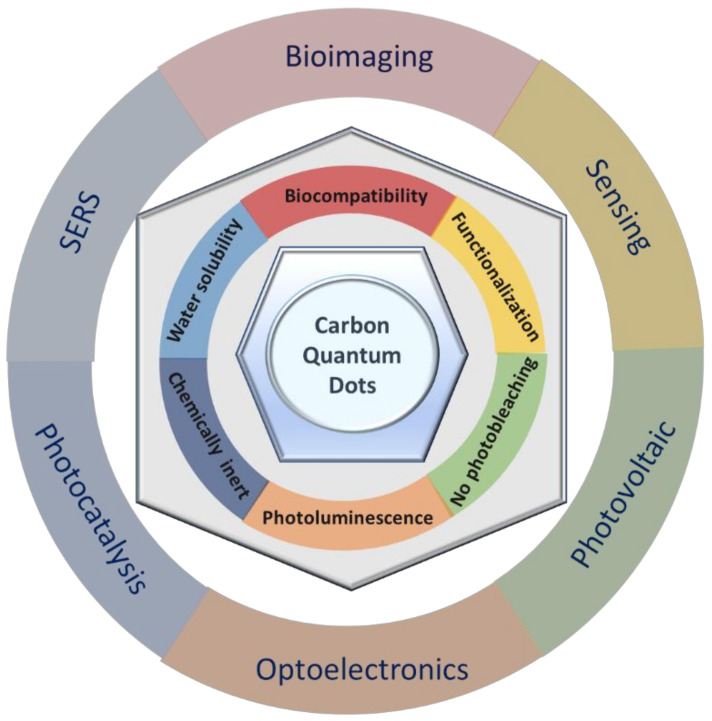
Schematic correlation between CQDs’ physical and chemical properties and possible applications in various fields.

Considering the described features and optical properties, CQDs have been employed in a wide array of applications ranging from electrochemical applications, wastewater treatment, bioimaging, and biotechnology, as well as chemical sensing and optoelectronic devices like LEDs. A small number of the possible applications are represented in Figure 10.

Additionally, in Table 2, Table 3, Table 4, Table 5, Table 6 and Table 7, one can find a literature review focused on the latest state of the art (between 2018 and 2020) of CQDs in terms of their diverse applications, to provide the readers with the present potential of CQDs in fields such as biomedical (Table 2), chemical sensing (Table 3), optoelectronics (Table 4), photocatalysis (Table 5), antimicrobial and antiviral (Table 6), and finally, other applications (Table 7). These tables include valuable information such as details of the synthesis methodologies, namely the starting materials employed and process conditions, the characterization and purification methods exploited and their impact in the final quality of CQDs, and the regular surface modifications and heteroatom doping used often to boost the target application of CQDs.

### 6.1. Biomedical

#### 6.1.1. Bioimaging

Fluorescence-based bioimaging has been sparking interest due mostly to its capacity for full-color imaging coupled with exceptional sensitivity (Table 3). Considering the systems in which such a platform is to be employed, the requirements for fluorescence imaging agents generally comprise low to inexistent toxicity, water solubility, and adequate physical/chemical properties. Thus, CQDs are the ideal nanosystem to succeed metallic semi-conductor QDs, generally regarded as quite toxic [200].

In 2019, Zheng et al. [118] synthesized CQDs through a one-pot synthesis using ammonium citrate dibasic mixed with either 3-aminophenylboronic acid, vancomycin hydrochloride, or polymyxin B sulfate to produce BA-CQDs, Van-CQDs, and PM-CQDs. The as-synthesized CQDs were then employed in the imaging and identification of six bacteria species: *Escherichia coli* (*E coli.*), *Desulfovibrio desulfuricans* (*D. desulfuricans*), *Staphylococcus sciuri* (*S. sciuri*), *Listeria monocytogenes* (*L. monocytogenes*), *Staphylococcus aureus* (*S. aureus*), and *Pseudomonas aeruginosa* (*P. aeruginosa*). The three recognition molecules utilized in CQDs synthesis could bind to all the bacteria with different abilities due to the variety of physicochemical nature of the different bacteria surfaces. Boronic acid CQDs presented the boronic acid ability to interact with *cis*-diol molecules present on the cell wall of bacteria, polymyxin CQDs manifested high affinity to lipopolysaccharide (LPS) molecules on the surface of bacteria, and vancomycin derived CQDs bonded specifically to (D)-Ala-(D)-Ala peptide found in the peptidoglycan layer on bacterial cell walls. Thus, using the mathematical statistical method “linear discriminant analysis” (LDA), the fluorescence pattern resulting from the mentioned interactions provided a good discrimination of the six kinds of bacteria with 91.6% correct accuracy and the classification of the bacteria according to the gram status of bacteria. Yang et al. [119] prepared CQDs via hydrothermal method from quercetin and ethylenediamine. The CQDs displayed pH-sensitive fluorescence effect under acidic and alkaline conditions and were, therefore, applied as a fluorescent sensor to monitor intracellular pH. The “OFF–ON–OFF” detection of pH values was based on the quenching of CQDs native fluorescence triggered by pH-induced aggregation. The fluorometric assay was also utilized for the detection of aspartic acid and glutamic acid, based on their isoelectric points. In 2020, Zhong et al. [120] produced blood compatible N,S-CQDs by hydrothermal synthesis from *m*-phenylenediamine and tobias acid. Characterization studies revealed a QY of 37.2%. In vitro studies were later performed to establish N,S-CQDs cytotoxicity towards human umbilical vein endothelial cells (HUVECs). Both confocal microscopy and flow analysis verified that N,S-CDs were efficiently taken up by HUVECs and were afterward localized in the cytoplasm, without considerable cytotoxicity. Additionally, hemolysis rates were determined to be inferior to 0.5% after exposure to different N,S-CQDs, suggesting blood compatibility. Shi et al. [121] reported the fabrication of red emissive CQDs from *p*-phenylenediamine by hydrothermal synthesis. The as-prepared CQDs were utilized as fluorescent nanoprobes for real-time sensing polarity in living cells based on the red shift of fluorescence emission wavelength verified in the presence of increasing polarity. The photoluminescence color of the CQDs varied from 525 to 612 nm in solvents with increasing polarity, and an 11-fold decrease in fluorescence intensity was verified when the solvent changed from 10% to 99% water. Moreover, the polarity assay was applied to different cell lines for the evaluation of intracellular polarity. Additionally, the CQDs were able to distinguish cancer cells from normal cells and displayed dual targetability both to mitochondria and lysosomes.

#### 6.1.2. Drug Delivery

A major requirement for safe drug delivery nano-vehicles is biocompatibility. Although in the past nano-drug delivery systems (DDSs) made use of chalcogenide-based quantum dots (QDs), toxicity and photobleaching presented themselves as impediments to this kind of nanosystem. Consequently, an alternative was needed, and considering CQDs with innate biocompatibility and properties, they have been extensively exploited in drug delivery systems, particularly those that combine both diagnostic and therapeutic capabilities [201,202]. Additionally, CQDs exhibit a high modification capability, a feature relevant considering that cellular internalization of drug delivery systems is improved by active targeting, for example, by promoting the interaction between overly expressed receptors on a cell surface and the targeting ligands that were incorporated on CQDs surface. Still, such an approach is not without flaws, and early clearance of CQDs from the bloodstream, causing a decrease in CQDs half-life, may occur due to recognition of target ligands by the mononuclear phagocyte system (MPS) or direct opsonization. Therefore, changes in the extracellular environment such as pH variations and altered enzyme expression can be the backbone for a triggered targeting approach, in which the target ligands are exposed by the effect of those changes in the extracellular environment, favoring the interaction with the desired receptors on the target cell [201].

**Figure 11:**
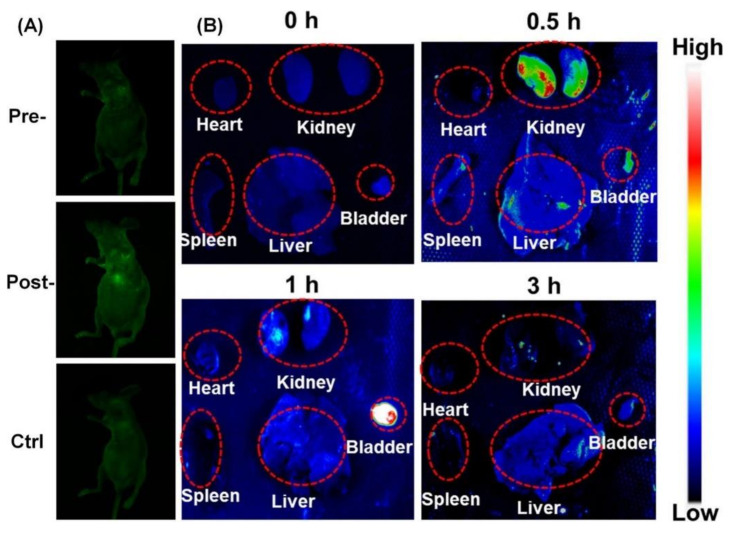
(**A**) In vivo imaging and (**B**) ex vivo imaging of a mice after intravenous injection of CQDs. Reproduced with permission from ref. [122], published by Springer Open, 2020.

Table 2 contains summarized a compilation of some works in which CQDsS were used and drug delivery vehicles. Sun et al. [122] obtained CQDs using sodium citrate dehydrate and urea by a one-step controlled thermal pyrolysis. Afterward, doxorubicin was non-noncovalently conjugated with CQDs by continuous stirring for 48 h in the dark. The DOX-CQDs were then capable of acid-triggered intracellular release of doxorubicin upon intracellular pH value changes, reacting to endo-/lysosomal pH to release the DOX within the cells in vitro and in vivo (Figure 11). Additionally, due to DOX-CQDs optical properties, the release of DOX was monitored, demonstrating inhibition to the proliferation of HO-8910 cells. In vivo, long-term toxicity tests did not reveal significant toxic effects in DOX-CQDs treated mice.

In 2020, Li et al. [123] prepared hyaluronic acid-modified CQDs (HA-CQDs) by one-step hydrothermal synthesis. Citric acid and branch-PEI were used as core carbon sources. The as-synthesized CQDs were then functionalized with hyaluronic acid for CD44 targeting (HA-CQDs). Afterward, doxorubicin was loaded onto the HA-CQDs, forming HA-CQDs@p-CBA-DOX through an acid cleavable bond, which caused drug-release in a pH-responsive manner. HA-CQDs@p-CBA-DOX exhibited good hemocompatibility and serum stability in in vitro experiments, while displaying elevated cytotoxicity on the target cells, 4T1 cells. The HA-CQDs@p-CBA-DOX was internalized by 4T1 cells through a hyaluronic acid-mediated CD44-targeting effect, leading to an augmented in vivo tumor accumulation of HA-CQDs@p-CBA-DOX, which was monitored by live imaging. Duan et al. [124] synthesized hyaluronic acid and heparin functionalized CQDs for the design of a drug delivery system, to target and inhibit the growth and migration of cancerous cells. The plain CQDs were obtained by dissolving glucose in a hydroglycolic solution, and after an initial hydrothermal treatment, PEI solution was added, and the hydrothermal treatment resumed. Then, hyaluronic acid and heparin were immobilized on the surface of CQDs via EDC/EHS coupling. Then, to obtain CQDs-HA-Hep/DOX, doxorubicin solutions at different concentrations were mixed with CQDs-HA-Hep solution at pH 7.4. The developed nanosystem was evaluated for in vitro drug release curve, blood compatibility, and cell viability, displaying blood compatibility with active targeting and release.

Hettiarachchi et al. [125] designed a triple conjugated system with ultrasmall CQDs with an average size of 1.5–1.7 nm. CQDs were prepared using carbon nanopowder mixed with sulfuric acid and nitric acid by reflux method. Then, CQDs conjugated with transferrin (the targeted ligand) and two anti-cancer drugs, epirubicin and temozolomide, by EDC/EHS coupling. The average size of the obtained nanosystem was only 3.5 nm, allowing efficient blood–brain barrier crossing. In vitro studies were performed with the cell lines SJGBM2, CHLA266, CHLA200 (pediatric), and U87 (adult) to assess the efficacy of the triple conjugated system compared to dual conjugated systems, non-transferrin C-dots-drugs, and free drug combinations. It was demonstrated that the triple conjugated system not only increased the cytotoxicity, but also the two-drug combination displayed the synergistic effect that caused 86% cytotoxicity to SJGBM2 cells.

#### 6.1.3. Gene Therapy/Delivery

Gene therapy relies on the treatment of both acquired and inherited genetic disorder diseases, by transporting genetic material into target cells to obtain either the augmentation of gene expression (as in DNA transfection) or the inhibition of target proteins by utilizing mechanisms such as small interfering RNA (siRNA) transfection. However, the physical and chemical nature of nucleic acids, mainly their fragility towards nucleases and their polyanionic nature that complicates cell approaching due to the negative potential of the cell membrane surface, calls for adequate carriers. While viral vectors provide for superior efficiency of gene delivery, the mass-production of these systems is not yet feasible. Therefore, the development of CQDs as non-viral carriers for gene delivery has been entailed, since it overcomes uneconomical and ineffective preparation and does not rely on scarce or intrinsically toxic elements [203]. Plus, the usage of CQDs in gene therapy provides the opportunity to follow the process of gene delivery, since it allows for the direct tracking of the fluorescent nanosystems, due to the intrinsic optical properties of CQDs [204].

Table 2 contains the most important aspects of some works found in the literature describing the use of CQDs for gene therapy or delivery. Shu et al. [126] developed a combination of CQDs/HER3 siRNA and trastuzumab for dual-targeted therapy in HER2-positive breast cancer cells, to overcome resistance to trastuzumab monotherapy. The CQDs were manufactured from citric acid monohydrate and ethanediamine through hydrothermal synthesis and were functionalized with branched PEI by EDC/EHS coupling. The resulting CQDs were positively charged, which allowed for efficient HER3 siRNA oligomer loading via electrostatic adsorption, forming CQDs/HER3 siRNA complexes. The transfection of CQDs and HER3 siRNA complexes caused the downregulation of HER3 protein expression, triggering considerable cell growth inhibition in BT-474 cells. Either CQDs/HER3 siRNA complexes or trastuzumab individually induced G0/G1 cell cycle arrest, but the combined treatment of CQDs/HER3 siRNA complexes and trastuzumab promoted much-marked cell growth suppression in BT-474 cells. Chen et al. [129] produced a CQDs based multifunctional gene delivery system with CQDs prepared by hydrothermal treatment of PEI 600 dissolved in ethanol. The obtained CQDs were modified with various hydrophobic chains and distinct degrees of substitution resorting to an epoxide ring-opening reaction. Then, the CQDs were employed as dual-functional non-viral gene vectors, in which the type and degree of substitution of the hydrophobic chain had a major impact on CQDs transfection efficiency. The as-prepared CQDs provided the advantage of being considerably less cytotoxic and presenting a better serum tolerance than classic cationic polymeric gene vectors. Ju et al. [130] synthesized CQDs for the specific inhibition of viral miRNAs by CQDs-mediated delivery of locked nucleic acid (LNA)-based suppressors. The CQDs were obtained by a microwave-assisted pyrolysis of citric acid and branched polyethyleneimine (PEI). Then, CQDs/LNA complexes were formed through electrostatic interaction between CQDs, which were positively charged, and the negatively charged LNA. The combination of CQDs/LNA caused the knockdown of KSHV miR-K1, miR-K4, and miR-K11 levels, inducing apoptosis and proliferation inhibition of KSHV-positive lymphoma cells by the activation of cleaved caspase 3. Additionally, effective inhibition of the initiation of the primary effusion lymphoma in the mouse model was accomplished, as well as induction of tumor regression, greatly improving survival rates.

#### 6.1.4. Others

Besides the previous biomedical applications, other examples closely related can be found in the literature, and these are included in Table 2. Jin et al. [127] produced CQDs using ascorbic acid and polyethyleneimine (PEI) by a one-step microwave-assisted method to evaluate the synthesized CQDs ability to affect pre-osteoblasts and determine the mechanism of the effects in vitro and in vivo. Osteogenic differentiation experiments and bone regeneration assays in vivo revealed that not only did CQDs increase the expression of bone-related factors, BSP and OCN, promoting pre-osteoblast differentiation in vitro, but they were also responsible for the promotion of bone regeneration in vivo. Pre-osteoblast differentiation was correlated to intracellular calcium increase, which leads to the induction of ER stress that caused the activation of PERK-eIF2α ATF4 signaling pathway, promoting the up-regulation of transcription factor ATF4, causing the increase in bone-related factors. In 2018, Malishev et al. [128] demonstrated for the first time the effect of chiral CQDs upon the aggregation process of amyloid beta-42 (Aβ42), a major constituent of amyloid plaques. CQDs were prepared either from L-lysine or D-lysine by a single-step carbonization procedure. The chiral CQDs, especially L-Lys-CQDs, presented high affinity to Aβ42 in both the monomeric and/or pre-fibrillar species and generated a modulation in the process of fibril assembly of Aβ42, interfered with their membrane interactions, and diminished the cytotoxicity of Aβ42. Wu et al. [144] designed a two-photon fluorescent probe composed of red emissive CQDs, graphene oxide (GO), and azobenzene (Azo) incorporated DNA (DNA-Azo) for in-situ imaging of miR-9 in Alzheimer’s disease mice living neurons and brains tissues. The prepared fluorescent probe was highly selective and sensitive towards miR-9, exhibiting a limit of detection of 0.57 fm. Moreover, it was revealed that miR-9 suffered up-regulation in Alzheimer’s disease of early-onset and that it was down-regulated to lower-than-normal levels in the late-onset of Alzheimer’s disease. Additionally, the two-photon fluorescent probe was tested as an alternative approach for imaging other miRNAs and medical pathological observation in ex-vivo biopsy.

### 6.2. Chemical Sensing

CQDs are recognized as exceptional sensors for both electrophilic and nucleophilic substances due to their extremely effective charge transfer quenching of fluorescence emission [205].

The application of CQDs to chemical sensing, either of biological molecules or inorganic compounds, relies on photophysical mechanisms, such as Förster resonance energy transfer (FRET), photoinduced electron transfer (PET), metal−ligand charge transfer (MLCT), electronic energy transfer (EET), intramolecular charge transfer (ICT), or twisted intramolecular charge transfer (TICT) [206]. Generally, the interaction between CQDs and the target compound is crucial to the sensing process, and as such, the distance between these two components of the process and complementary geometry are two parameters that directly impact the efficiency of the procedure.

Besides the referred photophysical mechanisms, the inner filter effect (IFE) can also be utilized to avoid some practical restrictions that may arise in those fluorescent assays. This process is prompted by the absorption of either exciting and/or emitted light by absorbers, which leads to an augmentation of sensitivity, since there is an exponential correlation between changes in the absorbance of sensors and changes in fluorescence intensity [207].

In Table 4 can be found the compilation of important data related to works found in the scientific literature, as examples of the chemical sensing application of CQDs, for biological molecules and ion sensing. These will be better discussed in the following.

#### 6.2.1. Biological Molecules

**Figure 12:**
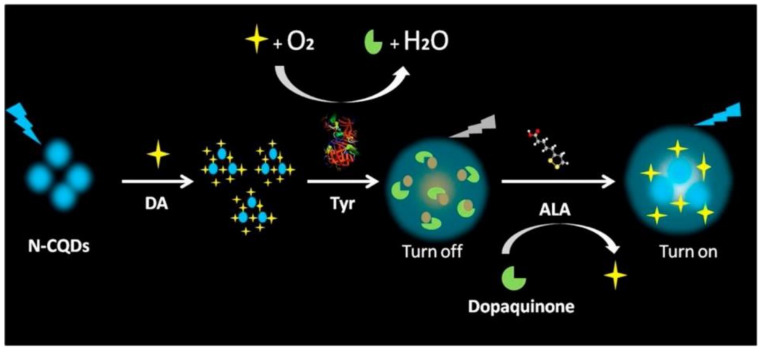
Underlying principle for the sensing of dopamine and alpha lipoic acid. Reprinted from ref. [147], with permission from Elsevier.

Zhang et al. [145] produced nitrogen-doped carbon dots (N-CQDs) for the determination of thiourea by a one-step hydrothermal method, using ammonium citrate and dextrin as precursors. Then, a known volume of Hg^2+^ in Britton–Robinson (BR) buffer solution was added to N-CQDs, triggering N-CQDs’ fluorescence quenching. However, the addition of thiourea promoted fluorescence recovery, since the interaction between thiourea and Hg^2+^ is stronger, therefore producing a fluorescent sensor based on the reversion of fluorescence quenching. Recovered fluorescence and concentration of thiourea exhibited a good linear correlation within the range of 0.90–10.0 mmol L^−1^. Thiourea determination was performed in tap water, lake water, and rice flour products with a detection limit of 0.15 mmol L^−1^. In 2020, Ma et al. [146] have developed B-doped CQDs (B-CQDs) as a signal–OFF–ON probe for catechol and glutathione detection. Catechol determination was based on fluorescence quenching, whereas glutathione sensing was achieved through fluorescence recovery without the need for auxiliary reagents. B-CQDs were manufactured through hydrothermal synthesis from citric acid and sodium tetraphenylborate and exhibited a QY of 42%. The linear range for catechol was 1–50 nmol L^−1^ and the detection limit was 0.25 nmol L^−1^, under optimum conditions. For glutathione sensing, the linear range was 2–100 nmol L^−1^ and a detection limit of 0.5 nmol L^−1^. The developed methodology displayed high selectivity for both catechol and glutathione sensing, even in the presence of a high concentration of interferents (biological thiols and amino acids). Thus, B-CQDs were employed in the detection of catechol and glutathione in river water and human serum, respectively. Wang et al. [147] developed a chemiluminescence (CL) system comprised of lucigenin (LUC) and CQDs for the detection of L-cysteine (Cys). The CQDs were prepared via hydrothermal treatment of starch and exhibited an absorption peak at 260 nm and a fluorescence emission peak centered at 524 nm (photo-excited at 470 nm). While the lucigenin–H_2_O_2_ CL system is very efficient, some disadvantages can be pointed out, such as insufficient stability and poor selectivity. Thus, CQDs were incorporated in the system, causing a strong enhancement of CL of lucigenin in alkaline media, while still retaining lucigenin as the luminophore. The enhancement was attributed to CQDs operation as electron donors, reacting with dissolved oxygen originating the superoxide anion radical (O_2_^•−^) in an alkaline medium, which in turn will react with Luc^•+^, forming lucigenin dioxetane (LucO_2_) that will be decomposed to excited NMA*, causing light emission from the transition of NMA* to NMA. L-cysteine enhances blue CL of the developed system linearly in the 10.0 to 100 μmol L^−1^ Cys concentration range, and a detection limit of 8.8 μmol L^−1^ was revealed, allowing for the sensitive determination of L-cysteine. The developed system was then used as a probe for dopamine and alpha lipoic acid, through the mechanism represented in Figure 12.

Zhang et al. [148] developed the first method resorting to spectroscopy to analyze alpha-lipoic acid (ALA) in real samples. This methodology was also used to determine dopamine (DA). The produced fluorescent nanosensor consisted of nitrogen-doped carbon quantum dots (N-CQDs) and tyrosinase, due to fluorescence emission and effective catalytic oxidation, respectively. The N-CQDs were prepared through hydrothermal synthesis, using anhydrous citric acid and ethylenediamine as starting materials. The as-synthesized N-CQDs displayed excitation independent behavior. The fluorometric assay was based on the quenching of N-CQDs’ native fluorescence caused by the addition of DA, and the recovery of said fluorescence by ALA, prompted by inhibition of DA oxidation. The developed “turn OFF–ON” assay for DA and ALA allowed a linear response between 0.05–15 and 0.5–55 μmol L^−1^, with a detection limit of 0.035 and 0.39 μmol L^−1^, correspondently. The quantification of DA and ALA in real samples was performed on human serum and urine samples, with an average recovery of 96–102% and 99–101%, respectively.

#### 6.2.2. Ion Sensing

Yue et al. [149] synthesized ethylenebis(oxyethylenenitrilo)tetraacetic acid (EGTA) modified CQDs via a two-step hydrothermal synthesis to determine intracellular calcium concentration. CQDs synthesis was achieved using citric acid and ethylenediamine as carbon and nitrogen sources, correspondingly. Afterward, the obtained CQDs were modified by the addition of EGTA, followed by a second hydrothermal treatment. A fluorescence platform was developed by correlating the decrease in CQDs fluorescence in the presence of increasing concentrations of calcium, caused by a static fluorescence quenching. This effect was then exploited for the detection of intracellular calcium in HeLa cell cultures, with linearity in the range of 15–300 μmol L^−1^ and a detection limit of 0.38 μmol L^−1^. Moreover, cytotoxicity assays were performed and revealed that CQDs only caused negligible toxicity, as cell viability was 90% at a concentration of 400 μg mL^−1^. Thus, the prepared CQDs were biocompatible and non-toxic. Singh et al. [150] produced CQDs utilizing mango (*Mangifera indica*: *M. indica*) leaves as starting material by pyrolysis treatment. The resulting CQDs had a QY of 18.2% and were applied to Fe^2+^ ion determination in both water and a Livogen tablet, which was used to validate the efficacy of the methodology, as it is a real sample. The CQDs displayed outstanding binding capability to Fe^2+^ ions over a wide range of pH values, most likely due to carboxylic and amine functional groups present in CQDs surface, which caused CQDs fluorescence to suffer quenching. The calculated detection limit was 0.62 ppm. Ali et al. [151] developed a CQDs based fluorometric assay for the determination of Cu^2+^ and S^2−^ ions in complex matrices. The CQDs were obtained by thermolysis of grape (*Vitis vinifera*) juice and presented a QY value of 32.1%. Afterward, CQDs were employed as dual-functional fluorescent probes for quantification of Cu^2+^ and S^2−^ ions in environmental water samples, based on the CQDs fluorescence quenching when in the presence of Cu^2+^, which can be caused by their adsorption on the CQDs surface. Upon adding S^2−^ ions, the fluorescence is recovered due to the stronger interaction between Cu^2+^ and S^2−^, which causes the desorption of Cu^2+^ ions from CQDs surface. The linear range was determined to be 0.07–60 μmol L^−1^ of Cu^2+^, with a limit of detection and a limit of quantification of 0.02 and 0.066 μmol L^−1^, correspondingly. As for S^2−^ ion detection, linearity exists in the range of 0.8 to 95 μmol L^−1^ with a limit of detection and a limit of quantification of 0.24 and 0.79 μmol L^−1^, respectively. Sinha et al. [152] fabricated a Cr^6+^ and Fe^3+^ ion sensing nanoprobe using CQDs based on CQDs fluorescence intensity quenching. The CQDs were obtained via hydrothermal synthesis using potatoes and exhibited a QY of 6.08%. The obtained CQDs were then utilized in a simultaneous and selective detection of Cr^6+^ and Fe^3+^ ions, even when other cations were present, in river water and tannery water samples. The linear range of the developed methodology was of 0.5–100 and 0.5–5 μmol L^−1^ for Cr^6+^ and Fe^3+^ ions. The detection limit was 0.012 and 0.000549 μmol L^−1^, correspondingly, which represents a concentration value inferior to the one specified by the World Health Organization (WHO).

### 6.3. Optoelectronics

Amongst the distinct optoelectronic applications that CQDs have been employed in, their inclusion in light-emitting diodes (LEDs) and solar cells of solar concentrators has been proving the remarkable adaptability of CQDs to several functions. However, aiming at meeting the requirements for CQDs insertion in photoelectronic devices implies that the water solubility that generally characterizes these nanomaterials must be substituted with affinity for organic solvents. Furthermore, the broad emission with the FWHM exceeding 80 nm is another feature frequently displayed by CQDs that must be avoided in photoelectronic devices. While metallic semi-conductor QDs usually present these properties more readily than CQDsS, they suffer from optical scintillation and manifest photobleaching, which in long term reduces the efficiency of LEDs [208]. When mentioning solar concentrators, environmental sustainability is a concern that leads to the exploration of the inclusion of “green” materials in those systems. Despite CQDs excellent properties, a limitation to be overcome is the low short-circuit current density (Jsc) presented by CQDs, which restricts the efficiency of the solar cells, comparatively with the open-circuit voltage (Voc) and fill factor (FF) of the metallic semi-conductor QDs, which begin to be commercially available [209].

#### 6.3.1. Light-Emitting Diodes (LEDs)

Table 5A summarizes examples of Carbon Quantum Dots used for LEDs. Yuan et al. [166] engineered triangular CQDs with narrow bandwidth emission via solvothermal treatment using three-fold symmetric phloroglucinol (PG) as the reagent (a triangulogen). The triangulogen was selected as a starting material, since it has an exceptional structure, with three extremely reactive hydrogen atoms at the three meta-positions that can be activated by three electron-donating hydroxyl groups, all in a single molecule. To obtain CQDs with narrow bandwidth emission, the triangular form was chosen, since this structure causes the weakening of the electron–phonon coupling and decreases surface defects that seem to be the primary origin of the broadband fluorescence, other than CQDs size polydispersity. After characterization, the as-synthesized CQDs were applied in blue to red LED fabrication, with CQDs imbued in the active emission layer. The LEDs were very stable and displayed high color-purity (FWHM of 30–39 nm), a L_max_ of 1882–4762 cd m^−2^, and η_c_ of 1.22–5.11 cd A^−1^. Guner et al. [167] synthesized N-CQDs for white-LED applications. The CQDs were prepared from diammonium hydrogen citrate and urea by reflux method. Then the as-produced CQDs were integrated in a PVPc composite film, originating drop-cast PVP/N-CQDs composite films. Higher concentrations of CQDs in the PVP/N-CQDs composite films generated white-light properties. Since the white light thus produced exhibits red deficiency, commercial inorganic red phosphors were included into PVP/N-CQDs composite films. Additionally, PVP/N-CQDs composite fiber were prepared and used as color conversion layers over a blue LED chip. Paulo-Mirasol et al. [168] studied the effect of surface ligands in inverted hybrid light-emitting diodes using CQDs. The CQDs were manufactured through either hydrothermal or hot injection methods. The hydrothermal synthesis of CQDs utilized citric acid, *p*-phenylenediamine (PDA) and ethylenediamine (EDA), or citric acid urea dissolved in dimethylformamide, depending on the desired capping ligand. Hot injection synthesis of CQDs capped by 1-hexadecylamine (HAD) was performed accordingly to a previously reported method [210,211]. It was verified that the usage of capped CQDs enhanced device performance when employed as hole transport layer. The nature of the selected capping ligand has a direct influence on the optoelectronic properties of CQDs, and it was observed that PDA created steric hindrance, which compromised the quality of the interface by inadequately covering the surface of the particle. Additionally, PDA-capped CQDs displayed a lower QY, resulting in devices with poorer current efficiency and luminance. However, employing CQDs capped with EDA generates a low turn-on voltage of 4.90 V, and regardless of the luminance of 70 cd/m^2^, CQDs can be used as hole transport materials. In 2020, Zheng et al. [169] fabricated CQDs through a one-step solvothermal method using 2,7-dihydroxynaphthalene and ethylenediamine as the carbon and nitrogen sources, respectively. The CQDs displayed an excellent QY value of 62.98%. 2,7-dihydroxynaphthalen was selected as the carbon source due to its benzenic ring and conjugated structure, which caused an increase in the electronic cloud density, originating CQDs with long-wavelength emission. To fabricate white light emitting diodes (white LEDs), the CQDs solution was dripped onto the inner wall of the optical lens and was dried in the oven at 80 °C for three hours. Afterward, the optical lens was fixed on the top of the LED chip. The produced white LEDs displayed a correlated color temperature of 2520 K and a color rendering index of 87, making them suitable for indoor lighting.

#### 6.3.2. Solar Energy Conversion

**Figure 13:**
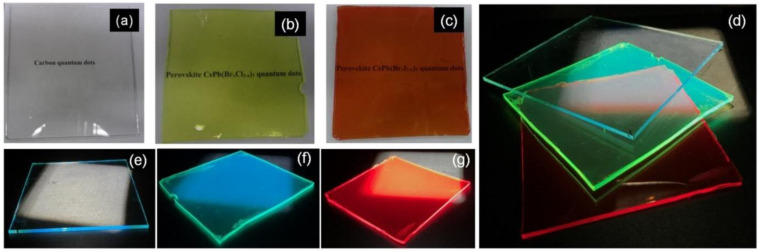
Photographs of the prepared LSC under ambient illumination (**a**–**c**) and one Sun illumination, 100 mW/cm^2^ (**d**–**g**). Reprinted from ref. [172], with permission from Elsevier.

In Table 5B, examples can be found of Carbon Quantum Dots used for solar converters. Padmanathan et al. [171] developed hybrid carbon dots/titanium dioxide (CQDs/TiO_2_) photoelectrodes for application in dye-sensitized photovoltaic solar cells. The CQDs were synthesized via a facile one-step hydrothermal approach utilizing citric acid and urea. Then, CQDs/TiO_2_ composites were obtained by mixture of CQDs with bare TiO_2_ nanopowder, followed by ultrasonication. The resulting particles size ranged from 30 to 40 nm. CQDs/TiO2 samples displayed higher surface areas (112 m^2^/g) and pore size (30–35 nm) than bare TiO_2_, which has a direct impact on solar cell efficiency. The photoanode fabricated from CQDs/TiO_2_ presented a significative conversion efficiency (PCE) of 15.5%. Zhao et al. [172] have reported the fabrication of luminescent solar concentrators (LSC), as seen in Figure 13, based on the simultaneous utilization of CQDs and mixed-halide inorganic perovskite QDs.

The CQDs were prepared by a hydrothermal synthesis using citric acid and ethylenediamine or citric acid and tris(hydroxymethyl)methyl aminomethane (Tris) as starting materials. The CQDs possessed a large Stokes shift and a QY of 50%. Then, CQDs were combined with polyvinylpyrrolidone (PVP) and spin-coated on a glass substrate. Finally, the semi-transparent tandem LSC based on CQDs and perovskite quantum dots manifested an external optical efficiency of ~3.05% when submitted to one Sun illumination, signifying a 27% and 117% augmentation in efficiency over single layer LSCs manufactured utilizing the same experimental conditions. Wang et al. [173] developed a nanocomposite thin film based on N-CQDs for application in luminescent solar concentrators (LSC). N-CQDs were incorporated in PVP thin films and optical properties and performance of N-CQDs/PVP thin film based LSCs with varying layer numbers and N-CQDs concentrations were ascertained, concluding that when the LSC layer numbers were 7 (0.5 wt% N-CQDs), the values of V_oc_, J_sc_, η_opt_, and η were 0.48 V, 19.10 mA/cm^2^, 5.02%, and 4.97%, correspondingly. Zdražil et al. [212] fabricated a tandem LSC using blue, green, and red CQDs. The CQDs were synthesized via hydrothermal method, and the utilized starting materials were citric acid monohydrate and L-cysteine for blue CQDs; 3,4,9,10-tetranitroperylene powder mixed with 0.2 M NaOH-ethanol mixture for green CQDs; red CQDs were obtained from the conversion of green CQDs after the addition of 1 M NaOH-ethanol solution until the solution turned from green to red. The QY values for the synthesized blue, green, and red CQDs were 63%, 78%, and 77%, respectively. The CQDs were then incorporated in tandem LSC, which possessed external optical quantum efficiency of 2.3% and an elevated degree of transparency, revealing 83.4% transmittance.

### 6.4. Photocatalysis

Heterogeneous photocatalysis refers to a chemical process in which a solid is responsible for the catalysis of a reaction using ultraviolet, visible, or infrared radiation as an external energy source. Upon photoexcitation, there is a production of electrons and holes that are capable of inducing, correspondingly, either reductive or oxidative reaction routes. Such a process can be exploited for selective organic molecules synthesis, generation of energy vectors, or even the removal of chemical residues or eradication of pathogens in either water or air [213]. CQDs are often employed in photocatalysis processes (Table 6) due to their outstanding capacity for harvesting photons in the visible and UV spectrums and, as photoexcited CQDs, by being exceptional electron acceptors and donors [205].

**Figure 14:**
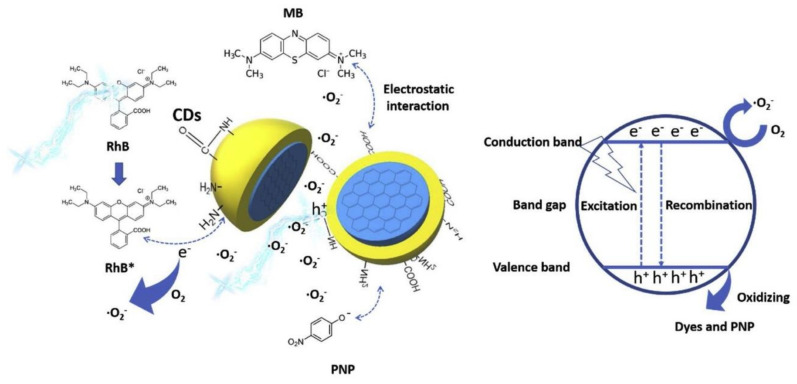
Schematic representation of the interaction between CQDs and dyes during photocatalytic degradation. Reprinted from ref. [114], with permission from Elsevier.

In 2019, Amadio et al. [180] tested the influence of carbon sources for CQDs in the photocatalytic activity and in structural properties. To do so, six types of CQDs were prepared either by hydrothermal or pyrolytic synthesis, generating, respectively, amorphous (-a) and graphitic (g-) CQDs, using citric acid, glucose, and fructose. It was verified that the easiness of CQDs formation is directly proportional to the reactivity of the carbon precursors towards decomposition/transformation. Thus, g-Fru-CQDs formed a multilayered supramolecular agglomerate of graphene-like crystalline sheets. It was also discovered that citric acid, as a carbon precursor, generated the best performing photoactive nanomaterials, followed by fructose and glucose derived nanomaterials. Moreover, the graphitization of the CQDs carbon core had a direct influence on their photoinduced electron transfer (PET) reactivity, which is fundamental in rational design of carbon-derived nano-photocatalysts. Zhou et al. [114] have induced photocatalytic degradation of both rhodamine B (RhB) and methylene blue (MB) using CQDs synthesized by microwave-assisted method and a mixture of citric acid and 1,2-phenylenediamine as precursors. The prepared CQDs did not show a quantum-size dependent fluorescence mechanism and thus manifested an excitation dependent emission behavior. After purification, three CQDs fraction were isolated and separately employed in the photocatalytic degradation of organic dyes under simulated sunlight irradiation, staying stable for multiple cycles. The interaction between the CQDs and the dyes is depicted in Figure 14. It was found that the photocatalytic activity of CQDs was inversely proportional to CQDs size. CQDs with an average size of 2 nm were capable of completely degrade both rhodamine B (RhB) and methylene blue (MB) in 150 min. The photocatalytic activity seems to be linked to holes and formation of superoxide radicals. Additionally, the 2 nm CQDs promoted photocatalytic degradation of p-nitrophenol (PNP).

Sabet et al. [178] synthesized N-doped CQDs via a simple hydrothermal, using grass as the starting material. The CQDs were used for the photocatalytic decomposition of six dyes, specifically Acid Blue, Acid Red, Methyl Orange, Eriochrome Black T, Methylene Blue, and Eosin Y, aiming to evaluate CQDs photocatalytic activity. Additionally, due to the high surface area of the N-CQDs, they were employed in Cd^2+^ and Pb^2+^ ions removal from water samples, since CQDs can adsorb the mentioned ions on their surfaces. CQDs adsorbed 37% and 75% of Cd^2+^ and Pb^2+^ ions, respectively. Zhu et al. [179] produced CQDs using waste palm powders as carbon source and thionyl chloride as the dopant, by a one-step hydrothermal method. The CQDs had an average size of 3.54 nm and an excitation dependent emission behavior. Then, the CQDs were used in the photocatalytic degradation of Rhodamine B and Methylene Blue due to their high photocatalytic activity, which was attributed to the S and Cl co-doped structure and small size effect. Under visible light, the Rhodamine B photoinduced degradation was nearly 71.7%, and Methylene Blue was 94.2% degraded, showing an almost complete degradation of the organic dye.

### 6.5. Antimicrobial and Antiviral Activity

Considering their chemical and optical properties, as well as natural biocompatibility, CQDs display a promising potential for antimicrobial and antiviral applications. CQDs antibacterial properties were found to be mainly dependent on the synthesis process, namely on the morphology and surface functional groups [214]. The presence of amine and amide functional groups on CQDs’ surface is known to cause an improvement in antibacterial activity, triggered by the electrostatic interactions established between CQDs and bacterial surfaces. Additionally, the generation of reactive oxygen species after adequate visible light irradiation on the bacterial surface seems to have a part in the antibacterial process. It should be noted that the absence of carboxyl, amino, hydroxyl, or sulfhydryl groups functional groups on CQDs surface is a motive for deficient electrostatic interactions with bacteria, conducing to inefficient antibacterial effects [215]. The same principles can most likely be also applied to other antimicrobial activities such as antifungal or antiparasitic. Nowadays, there is not an efficient and specific therapeutical arsenal or vaccines to combat some classes of virus (human or non-human), and since new viral emergencies are expected in the future, the necessity for classical antiviral treatment alternatives is more flagrant than ever. We currently have the coronavirus 2 (SARS-CoV-2) as an example, the strain of coronavirus that caused the coronavirus disease 2019 (COVID-19), responsible for a pandemic that is persisting for months. Additionally, other viral classes may start developing resistance to antiviral agents, reducing their clinical value. As such, investigation has been undertaken in the direction of exploring the antiviral activity of CQDs. CQDs are capable of, for example, inhibiting the binding of virus to host cell receptors [216] or preventing the multiplication of virus [217], which could be valuable when therapeutically options are scarce. Table 7 contains relevant information about some examples of CQDs used as antimicrobials and antivirals.

#### 6.5.1. Antimicrobial Activity

Travlou et al. [190] studied the relation between the incorporation of different heteroatoms into CQDs and the bactericidal activity presented by said CQDs. Sulfur and nitrogen-doped carbons quantum dots (S-CQDs and N-CQDs) were manufactured using poly (sodium-4-styrene sulfonate) and polyvinylpyrrolidone, respectively. Afterward, CQDs bactericidal activity was tested against both Gram-negative exemplary species (*Escherichia coli*, CECT 831) and Gram-positive (*Bacillus subtilis* subsp. *subtilis* 168). It was found that N-CQDs presented a greater bactericidal activity comparatively to S-CQDs and that CQDs surface chemistry and sizes had a direct impact on their activity. N-CQDs triggered bacterial death through a mechanism that involved the electrostatic interactions between their protonated forms and the lipids of the bacterial cell membrane and, possibly, by the activation of oxygen species by CQDs surface. Additionally, amides and amines were proven to have an important role in the bactericidal effect enhancement. S-CQDsS presented a size dependent action and contained a negatively charged surface due to dissociation of sulfonic/carboxylic groups and sulfates. Overall, the synthesized CQDs minimum inhibition concentrations were similar or smaller than those of some antibiotics or silver nanoparticles. Song et al. [191] obtained CQDs by pumping cigarette smoke through water, producing a brownish yellow solution of CQDs. Next, the antibacterial activity of the CQDs against *Escherichia coli*, *Staphylococcus aureus*, ampicillin-resistant *E coli.* (AREC), and kanamycin-resistant *E coli.* (KREC) was evaluated to be within the maximum concentration of 1000 μg mL^−1^. The mechanism for the bactericidal activity presented by CQDs is related to the destruction of double helix structure of DNA and does not cause alterations in the morphology of the bacteria. Additionally, the synthesized CQDs can be broken down into smaller particles and organic fragments in the presence of horseradish peroxidase (HRP) and H_2_O_2_ in seven days and still exhibit efficient antibacterial activity within the first 2–4 days. In addition, four other kinds of CQDs were prepared from camphor tree leaves, mulberry leaves, lalang grass rhizome, and *schizonepeta tenuifolia*, but were unable to inhibit bacterial growth, proving that starting material selection and consequently, the chemical composition of the synthesized CQDs, can have a prevailing effect upon CQDs’ practical application. In 2020, Muktha et al. [131] synthesized CQDs resorting to organic waste of pomegranate and watermelon peels in a microwave-assisted synthesis. The synthesized CQDs antimicrobial activity was tested against the ascomycete fungus *Fusarium oxysporum* and several bacteria, such as *Staphylococcus aureus*, *Pseudomonas aeruginosa*, *Bacillus subtilis*, and *Escherichia coli*. It was concluded that CQDs derived from pomegranate peel (P-C dots) have a more significative antimicrobial effect than those synthesized from the watermelon peel (W-C dots). P-C dots have also shown to have a strong antifungal activity when compared to fluconazole, while W-C dots did not manifest any antifungal effect. In addition, both P-C dots and W-C dots have manifested anti-cancer effects against MCF-7 and HepG2 cell lines. Li et al. [192] produced completely degradable and low-toxicity CQDs utilizing vitamin C as the carbon precursor through a one-step electrochemical synthesis. The CQDs can be totally degraded into CO_2_, CO, and H_2_O by irradiation of visible light by the action of very mild temperatures (about 37 °C) after 20 days. The CQDs were then tested for antibacterial activity against several bacteria, including *Staphylococcus aureus*, *Bacillus subtilis*, and *Escherichia coli* (non-resistant and ampicillin-resistant) and antifungal activity against *Rhizoctonia solani* and *Pyricularia grisea.* The best reported bacteriostatic and fungistatic concentrations were 100 and 300 μg mL^−1^, correspondingly. The suggested mechanism of action for CQDs was the entrance in either bacteria or fungus by diffusion, followed by the destruction of the microorganisms wall, binding to bacterial and fungal DNA and RNA and RsNPs gene expression inhibition, which ultimately lead to microbial death at low CQDs concentration.

#### 6.5.2. Antiviral Activity

**Figure 15:**
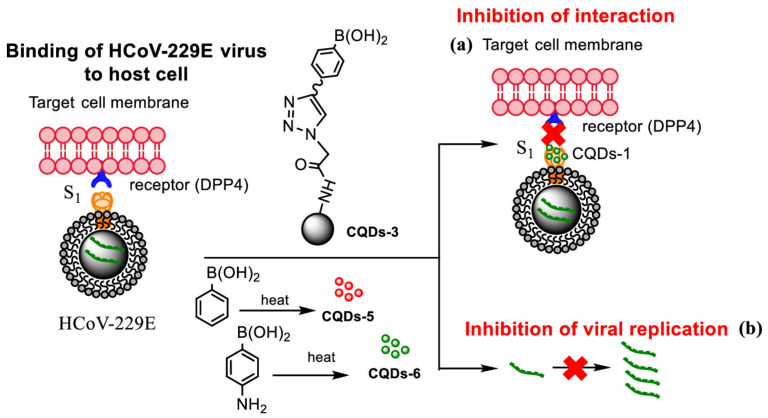
Proposed mechanisms for viral replication inhibition of HCoV-229E-Luc infection by CQDs, through the interference with protein S receptor interaction (a) and by the inhibitory influence on the replication of viral RNA genome. Reproduced with permission from ref. [193], published by American Chemical Society, 2019.

Łoczechin et al. [193] have evaluated the antiviral activity against human coronavirus HCoV229E of seven distinct types of CQDs. Three of the synthesized CQDs caused significant inhibition of HCoV-229E-Luc infection in a concentration-dependent manner, while a fourth type of CQDs manifested only a mild activity against the virus. However, all the seven types of CQDs interfere with the viral replication step. Mechanistic studies indicate that CQDs affect mainly the entry of the virus into the host cell, which could be caused by interference with protein S-receptor interaction with the host cell membrane, as depicted in Figure 15.

In 2019, Huang et al. [194] reported the design of CQDs derived from benzoxazine monomers (BZM-CQDs) and the in vitro evaluation of BZM-CQDs capacity for blockage of multiple viruses’ infectivity. The tested viruses were flaviviruses (dengue, Zika, and Japanese encephalitis viruses) and non-enveloped viruses (porcine parvovirus- and adenovirus-associated virus). It was discovered that BZM-CQDs were capable of direct interaction with virions and, consequently, caused the inhibition of virion entrance into the host cell. Due to this mechanism, it was concluded that BZM-CQDs might provide a broad-spectrum therapeutic possibility against phylogenetically related and unrelated viruses. Tong et al. [195] synthesized glycyrrhizic acid derived CQDs (Gly-CQDs) from the medicinal Chinese plant *Glycyrrhiza uralensis* employing the hydrothermal method. Said Gly-CQDs were later determined to possess an excellent antiviral activity (≈5 orders of magnitude) against porcine reproductive and respiratory syndrome (PRRSV). Additionally, mechanistic studies revealed the antiviral mechanism of Gly-CQDs to be based on the inhibition of PRRSV invasion and replication in vitro, inactivation of PRRSV, blockage of ROS production induced by PRRSV infection, and stimulation of host immune cells to increase interferon production. Proteomics studies suggested that Gly-CQDs are responsible for the upregulation of intracellular antiviral proteins and downregulation of pro-viral proteins. Huang et al. [196] evaluated the antiviral activity of polyamine-modified CQDs (polyamine CQDs) against white spot syndrome virus (WSSV), responsible for the white spot syndrome (WSS), which is a common cause of death of cultured shrimp. Polyamine CQDs could bind to the WSSV envelope, inhibiting the viral infection in a dose-dependent manner. Moreover, polyamine CQDs generated an upregulation of various immune genes in shrimp, including antimicrobial immune responses, hemolymph clotting mechanism, antioxidant defense mechanism, and antimicrobial peptide system, although the immune gene expression was not directly proportional to the dosage administered. Polyamine CQDs also caused a decrease in shrimp mortality upon WSSV infection.

## 7. Pharmacokinetics, Pharmacodynamics, and Toxicity

Despite the well-known water solubility and biocompatibility displayed by CQDs, their application on the clinical scenario depends on investigating possible toxicity pathways and meticulously study their pharmacokinetics. All implanted biomaterials have the potential to trigger immunological responses, such as foreign body responses (FBRs), known to cause an inefficient activity of CQDs [218]. They may also trigger unwanted biological interactions, such as bio-coronation by proteins, lipids, or other macromolecules. Furthermore, the possibility of enzymatic degradation by immune-competent cells should not be ignored, as it can compromise CQDs biological activity. Furthermore, with the intention of employing CQDs as therapeutic agents, a detailed exploration of their pharmacokinetic profiling concerning adsorption, distribution, metabolism, and excretion (ADME) must be undertaken. Finally, the role of metabolic activity on CQDs action in vivo is of outmost importance, as both bioactivation and biodegradation can lead to unwarranted effects [219].

**Figure 16:**
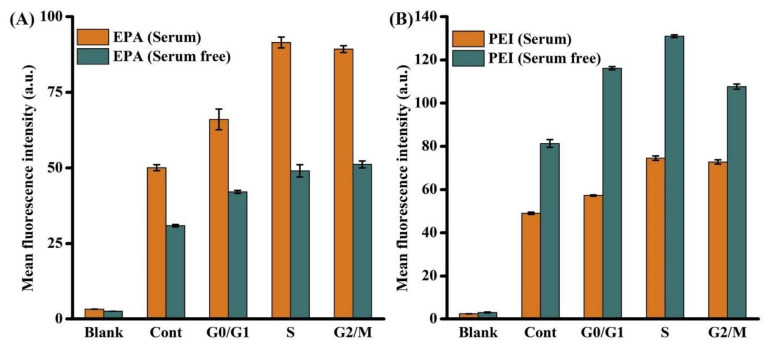
Intracellular uptake of EPA-CQDs (**A**) and PEI-CQDs (**B**) by normal HeLa cells (Cont) and by cells at three cell cycle phases (G0/G1, S, and G2/M), cultured in media with and without serum. Reprinted from ref. [222], with permission from Elsevier.

In 2019, Singh et al. [220] systematically evaluated the in vivo and in vitro toxicity of N-CQDs. For the first time, an extensive assessment of the phenomena of in vitro cyto- and genotoxicity of CQDs was entailed, and an in-depth analysis of hematological, biochemical, and histopathological parameters in vivo was performed in Swiss albino mice. The N-CQDs utilized for the study were synthesized from D-glucose and ethylenediamine that, after neutralization with acetic acid, were submitted to microwave irradiation. The as-prepared N-CQDs manifested an excitation dependent emission behavior and an average size of ∼3.7 nm. The N-CQDs also possessed a highly hydrophilic nature. The in vivo toxicological effect monitoring was carried out for up to 30 days, at two distinct N-CQDs concentration, namely 5.0 mg per kg body weight (BW) and 10.0 mg per kg BW. No acute morphological and toxicity changes were verified, and results of hematological, serum biochemical, antioxidant, and histopathological parameters did not imply significative N-CQDs toxicity. Furthermore, the liver, kidney, and spleen remained without observable inflammation or disruption at the 30th day after the administration of the N-CQDs. Finally, the in vitro assay was executed against HeLa cells, using N-CQDs in the concentration range of 0–400 µg mL^−1^. The growth cycle of cells, lactate dehydrogenase (LDH) profile, apoptosis, and DNA fragmentation corroborated the in vivo results. Thus, N-CQDs were deemed nontoxic via intravenous application route and up to the tested concentration of 400 μg mL^−1^. Liang et al. [221] have prepared three different types of nitrogen- and sulfur-doped CQDs (N,S-CQDs) to study their interaction with human serum albumin (HSA). The three kinds of CQDs were produced either using citric acid (CA), glucose, or ascorbic acid (AA) as distinct carbon sources, and the combination of urea and N-Acetyl-L-cysteine as nitrogen and sulfur dopants via hydrothermal method. The CQDs were named according to their carbon source, specifically CQDs (CA) for citric acid, CQDs (glucose) for glucose, and CQDs (AA) for ascorbic acid. Upon characterization, CQDs (glucose) and CQDs (AA) manifested excitation dependent emission behavior, while CQDs’ (CA) fluorescence was excitation independent. Additionally, all CQDs displayed a pH-dependent fluorescence and were highly stable under different ionic strengths and prolonged light exposure. However, CQDs fluorescence decreased upon the increase of temperature, revealing a weak thermostability. Concerning CQDs interaction with HSA, it was concluded that the different types of CQDs showed distinct quenching mechanisms on the intrinsic fluorescence of HSA. While both CQDs (glucose) and CQDs (CA) caused a decrease in HSA fluorescence by dynamic quenching, the interaction between CQDs (AA) caused static quenching. Considering CQDs (AA) quenching mechanism, site-selective binding with HSA was studied by using Warfarin, which binds to the site I of serum albumin, and ibuprofen, which binds to site II of serum albumin. The assay revealed that the CDs (AA) were mainly present in site I of HAS, leading to conclude that CQDs (AA) preferably interacted with site I of HSA, which is also the binding site for Warfarin. Finally, it was concluded that all CQDs could induce conformational changes in HSA. Yan et al. [222] have evaluated the intracellular uptake of two differently functionalized CQDs at three cell cycle phases (G0/G1, S and G2/M) using HeLa cells, as well as different internalization pathways and translocations. The CQDs were obtained by functionalization of CQDs synthesized from commercially acquired carbon nanopowders. EPA-CQDs and PEI-CQDs were produced functionalizing CQDs with 3-ethoxypropylamine and polyethylenimine, respectively. Concerning internalization pathways, a slightly higher internalization efficiency for PEI-CQDs was verified, and the presence of serum could effectively enhance cellular uptake of EPA-CQDs, while significantly inhibiting that of PEI-CQDs. The study of the effect of cell cycle phase on CQDs uptake revealed that when serum was present, the main uptake pathway of EPA-CQDs was clathrin-mediated endocytosis [223]. Additionally, caveolae-mediated endocytosis [224] was observed, particularly in cells at the S phase. In cells at the G1/G0 phase, there was also micropinocytosis. In serum-free media, EPA-CQDs suffered uptake mainly through clathrin-mediated endocytosis. As for PEI-CQDs, in serum-containing media, caveolae-mediated endocytosis and micropinocytosis were accountable for the some of the verified uptake, implying the involvement of other internalization pathways. In serum-free media, the uptake of PEI-CQDs was caused by the contribution of clathrin-mediated endocytosis, micropinocytosis, and caveolin-mediated endocytosis, in cells at S phase. In cells at G0/G1 phase, the uptake was triggered by clathrin-mediated endocytosis, with minor contribution by caveolae-mediated endocytosis, whereas in cells at G2/M, the only pathway involved was caveolae-mediated endocytosis, with a very small contribution, leading to the conclusion that the internalization of PEI-CQDs by the HeLa cells is strongly affected by cellular cycle. As for the intracellular localization of CQDs, it was found that while the lysosome was the only destination for EPA-CQDs, PEI-CQDs were initially found on both lysosome and mitochondria and then moved out of mitochondria, remaining in the lysosomes. Figure 16 illustrates the differences in the intracellular uptake of EPA-CDots and PEI-CDots.

In 2019, Srivastava et al. [225] described CQDs degradation kinetics after subjection to enzymatic oxidation, in an attempt at clarifying the metabolic fate of these nanosystems in a biological environment. CQDs were firstly manufactured via hydrothermal method using sucrose as the carbon source and polyethylene glycol (PEG), phospholipids, and polyethylene glycol phosphate, for the formation of CQDs-PEG, CQDs-lipid, and CQDs-PO_4_, respectively. Additionally, CQDs-PEI were obtained by functionalization with polyethyleneimine (PEI). Therefore, the passivation agent was responsible for the surface charge of CQDs, allowing the design of neutral, zwitterionic, positive, and negative CQDs corresponding to CQDs-PEG, CQDs-lipid, CQDs-PO_4_, and CQDs-PEI. Then, the as-prepared CQDs were submitted to peroxide catalyzed degradation in the presence of porcine lipase, which showed that not only the surface charge of CQDs promoted distinct degradation kinetics upon enzymatic oxidation, but also that their decomposition correlates with CQDs accessibility to porcine lipase. It was found that CQDs-PEG were the first CQDs to be degraded, followed by CQDs-PO_4_ and CQDs-PEI. The last CQDs to be degraded were CQDs-lipid, possibly due to the initial degradation of lipid molecules, which delayed the degradation of CQDs-lipid. Regarding the formed metabolites, hydroxymethylfurfural was a common metabolite produced after the degradation of all CQDs.

## 8. Conclusions and Future Perspective

Considering CQDs’ unique and versatile properties, it is not surprising that they have been extensively explored in diverse applications, resulting in an accentuated growth of the scientific publications related to the field of CQDs research in recent years. Amongst other useful properties, CQDs are known to possess low cytotoxicity, photochemical stability, and easily modified surface, which not only makes them formidable alternatives to metal-based semiconductor QDs, but also could revolutionize our understanding of several fields, especially biomedicine. The chemical and physical characteristics of CQDs that make them ideal for such a vast number of scientific thematic and fields of applications are mainly imparted during their synthesis. The innovation that has been experienced in CQDs synthesis, from intricate methods to greener synthesis using common sustainable organic materials, is partially responsible for the attention this kind of nanomaterial has garnered. Green synthesis of CQDs is a strategy that has allowed the production of CQDs without the need for environmental aggressive chemicals or substantial energy consumption due to high experimental temperatures, ensuing in a decrease in production cost and promoting the implementation of sustainable development in CQDs research field.

Nevertheless, despite the considerable collective work that has been entailed on the synthesis, purification or separation, application, and investigation of fluorescence mechanism, there is still much to be elucidated. Regarding CQDs synthesis, contemporary challenges present themselves in the lack of fine control of size, crystallinity, and morphology of CQDs, which is exposed by the shortage of clear-cut synthesis protocols for the manufacturing of well-defined chemical identities. Additionally, considering that intrinsic structure and surface components of CQDs have a direct impact on their fluorescence, low QY and non-uniform optical properties are not uncommon amongst this new nanomaterial, although some examples prove that this challenge can be overcome. Other than the difficulty in having a precise manipulation of CQDs properties and characteristics, the fact that the fluorescence mechanism of CQDs is yet to be established is another reason that could be responsible for low QY and related phenomena. Until the origin of CQDs fluorescence is documented and understood, the deficiency of intensive brightness could prove to be an obstacle to some applications, particularly bioimaging, in which high QY is crucial. To transport the experimental data to clinical reality, besides surmounting the previously mentioned challenges, the study of pharmacokinetics and biodistribution of CQDs must be profoundly ensued, as well as the multifaceted interaction between CQDs and biological environments. For that end, the interaction between CQDs and proteins, cells, or various types of tissues must be investigated.

Notwithstanding, tackling and vanquishing the present challenges and obstacles that are intertwined with the evolution of CQDs science will have a critical impact on the field. The uniqueness displayed by CQDs, even though much knowledge needs to be learned, is the driving force that will propel chemists, biologists, engineers, material scientists, and clinicians to find brand new ways to synthesize, understand, modify, and apply these nanomaterials that are singular in their biocompatibility, optical properties, as well as chemical and physical stability. Soon, should the clinical use of CQDs become a reality, the benefit for patients in diverse clinical situations and society in general will be, undoubtedly, palpable. At the dawn of a new decade, the possibilities for this new nanomaterial are almost limitless, and when the hindrances that block some of the CQDs potential have been scrutinized and further investigated, the CQDs research field will be bound to flourish.

## Figures and Tables

**Table 1:** Comparison of the different photoluminescence mechanisms and particular features of semiconductor quantum dots (SQDs) and carbon-based nanomaterials.

Type of Nanodot	SQDs	CQDs	GQDs	CNDs
**Fluorescence Scheme**	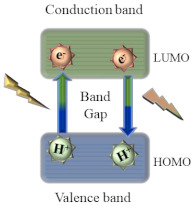	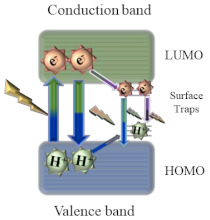		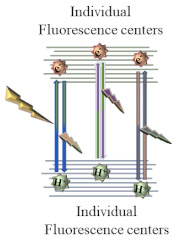
**Structure**	Inorganic elements in spherical crystalline structures	Mixture of sp^2^ and sp^3^ carbons in a quasi-spherical crystalline structure	Discal fragments of single nanosheets of sp^2^ carbons	Quasi-spherical amalgams of sp^3^ carbons in an amorphous structure
**Typical Size**	<6 nm	<10 nm	<20 nm	<10 nm
**Quantum Confinement**	Yes			No
Light Emission	Down-conversion PL Phosphorescence	Down-conversion PL Up-conversion PL	Down-conversion PL Up-conversion PL Phosphorescence	Down-conversion PL Up-conversion PL
	Size dependent PL	Size dependent PL (undefined)	Size dependent PL (undefined)	Size-dependent PL
	Narrow PL bands	Broad PL bands		Very broad PL bands
	Long PL lifetimes	Medium PL lifetimes		Short PL lifetimes

**Table 2:** Advantages and disadvantages for the most common CQDs synthesis methods.

Method	Advantages	Disadvantages	Ref.
Hydrothermal synthesis	Good production yields, ease of manipulation, good production yield of nanomaterials using high vapor pressure settings	Long synthesis duration	[21]
Microwave synthesis	Clean, ease of manipulation, low-temperature, and economic	Bulk metallic materials unusable due to electromagnetic field interferences	[22,23]
Electrochemical synthesis	Ease of operation, potential for mass production, does not involve harsh or toxic chemicals	Laborious purification processes	[24]
Sonochemistry synthesis	The only general method for doping CQDs using bulk metals (Ga, In, Bi, Sn, Pb, Cd, Sb, and Zn)	Prolonged synthesis process	[25]
Laser ablation synthesis	High quality CQDs, swift synthesis	Low reproducibility; difficulty in correlating experimental conditions with obtained CQDs properties	[26,27]
Biomass synthesis	Simple, cost effective, and easily available in nature	Possible environmental contamination; necessity of additional chemical modifications to increase QY	[16,28]

**Table 3:** Summarized examples of carbon quantum dots used for biomedical applications.

Application	Target	Ligands	Receptor	CQDs Synthesis Method	Precursors	Reaction Conditions	Modifications and Functionalization	Purification	Average Size (nm)	QY (%)	Ref.
Bioimaging	HeLa cells	n/a	n/a	Hydrothermal	Citric acid ethylenediamine alkali lignin	150 °C, 4 h	Lignin hybridized	Dialysis (3500 Da, 2 days)	<10	43	[30]
Bioimaging	Hela, SMMC-7721 and A549 cells	Folic acid	Folate receptors	Hydrothermal	Aconitic acid Ethylenediamine	150 °C, 5 h	N-doping	n/a	1.6	56.5	[31]
Growth promotion	Mung bean plant	n/a	n/a	Hydrothermal	Citric acid *L*-Cysteine *D*-Cysteine	180 °C, 1.5 h	N, S-doping Chirality	Dialysis (500–1000 Da, 5 days)	6	_L_-CDs: 31 _D_-CDs: 33	[32]
Bioimaging Antitumor therapy	HeLa cells (in vivo and in vitro)	Gallic acid	n/a	Microwave-assisted	Gallic acid Citric acid Ethane diamine	700 W, 3 min	Gallic acid hybridized	Centrifugation (10,000× *g* rpm, time 10 min) Dialysis (500 Da, 72 h)	2–4	25	[37]
Bioimaging	*Streptococcus* sp. and *Klebsiella pneumoniae*	n/a	n/a	Mechanical and ultrasonic millings	Curcumin (BOSF)	Pulsed mode (750 W, 20 kHz), intensity of 30 W/cm^2^, 20 min	n/a	n/a	13.7	n/a	[26]
Bioimaging	HeLa cells, L02 cells, and macrophage cells	n/a	n/a	Pulsed laser ablation	*Platanus* biomass	Repetition rate of 10 Hz, wavelength of 1064 nm, 20 mJ, 3–6 ns, 30 min	N-doping	Centrifugation (10,000× *g* rpm, time n/a) × 5	8	32.4	[50]
Bioimaging	*E. coli*, *Aspergillus aculeatus* and *Fomitopsis* sp.	n/a	n/a	Hydrothermal	*Manilkara zapota* fruits	blue C-dots: 100 °C, 60 min	n/a	Dialysis (12,000–14,000 Da, 24 h)	1.9	5.7	[52]
						green C-dots: 80 °C, 30 min			2.9	7.9	
						yellow C-dots: 80 °C, 15 min			4.5	5.2	
Bioimaging	SMMC7721 cells	n/a	n/a	Hydrothermal	*p*-Phenylenediamine Ammonia	200 °C, 8 h	N-doping	Dialysis (500–1000 Da, 3 days)	3.2	13.2	[57]
Bioimaging	HEp-2 cells	n/a	n/a	Hydrothermal	Citric acid Methionine	200 °C, 3 h	N, S-doping	Centrifugation (12,000× *g* rpm, 15 min) Dialysis (500 Da, 12 h)	5	13.8	[67]
Cellular ATP monitoring	T24 cells	n/a	n/a	Hydrothermal	*L*-Cysteine *D*-Cysteine	60 °C, 24 h	N, S-doping Chirality	Dialysis (MWCO n/a, time n/a)	5–7	41.26	[68]
Bioimaging Ion detection	MCF-7 cells	n/a	n/a	Microwave-assisted	1,6-Hexanediamine dihydrochloride Dimethylsulfoxide	180 °C, 35 min	N, S-doping	Filtration with filter membrane (0.22 μm) Dialysis (500 Da membrane,36 h)	4.35	24	[69]
Bioimaging	HeLa and MCF-7 cells	n/a	n/a	Hydrothermal	3-Aminobenzeneboronic acid 1,2-Ethylenediamine	160 °C, 7 h	N, B-doping	n/a	2.61	47.3	[72]
Bioimaging Bacteria identification	*Escherichia coli* (*E. coli*), *Desulfovibrio desulfuricans* (*D. desulfuricans*), *Staphylococcus sciuri* (*S. sciuri*), *Listeria monocytogenes* (*L. monocytogenes*), *Staphylococcus aureus* (*S. aureus*) and *Pseudomonas aeruginosa* (*P. aeruginosa*)	3-Aminophenylboronic acid/vancomycin hydrochloride/polymyxin B sulfate	cis-diol/(D)-Ala-(D)-Ala peptide/LPS	Hydrothermal	Ammonium citrate dibasic 3-Aminophenylboronic acid/vancomycin hydrochloride/polymyxin B sulfate	180 ℃, 4 h	3-Aminophenylboronic acid/vancomycin hydrochloride/polymyxin B sulfate hybridized	Centrifugation (10,000× *g* rpm, 15 min) Dialysis (1000 Da, overnight)	3–6	n/a	[118]
Intracellular pH imaging	HepG2 cells	n/a	n/a	Hydrothermal	Quercetin Ethylenediamine	200 °C, 12 h	N-doping	Filtration with filter membrane (0.22 μm) Silica gel column chromatography (CH2Cl2/MeOH (V/V, 20/1))	2.7	13.4	[119]
Bioimaging	HUVEC cells	n/a	n/a	Solvothermal	*m*-Phenylenediamine Tobias acid	200 °C, 10 h	N, S-doping	Centrifugation (10,000× *g* rpm, 10 min.) Filtration with filter membrane (0.22 μm)	2.59	37.2	[120]
Cell polarity imaging	HepG2 cells	n/a	n/a	Hydrothermal	*p*-Phenylenediamine	200 °C, 12 h	N-doping	Centrifugation (6577× *g*, 15 min) Silica gel column chromatography (EtOH/Ethyl acetate)	5	3.40 (in water)	[121]
Drug delivery Bioimaging	HO-8910 cells	Doxorubicin	n/a	Carbonization	Sodium citrate dehydrate Urea	200 °C, 1 h	N, S-doping Conjugation with doxorubicin	Centrifugation (10,000× *g* rpm, 10 min, 3 ×) Dialysis (1000 Da, 24 h)	2.75	93	[122]
Drug delivery Bioimaging	4T1 cells	Hyaluronic acid	CD44	Hydrothermal	Citric acid Branched-PEI	180 °C, 1 h	Hyaluronic acid-modified Conjugation with doxorubicin	Ultrafilytration (MWCO: 50,000), 4000 rpm, 20 min, 4×	10	n/a	[123]
Drug delivery Bioimaging	MGC-803 cells	Hyaluronic acid	CD44	Hydrothermal	Glucose Glycol + PEI	180 °C, 3 h + 80 °C, 3 h	Hyaluronic acid-modified Conjugation with heparin	Filtration with filter membrane (0.22 μm) Centrifugation (10,000× *g* rpm, 20 min)	3.9	n/a	[124]
Drug delivery Bioimaging	SJGBM2, CHLA266, CHLA200 and U87 cells	Tranferrin	Tranferrin receptors	Reflux	Carbon nano powder	110 °C, 15 hs	Triple-conjugated system (epirubicin, temozolomide and transferrin)	Centrifugation (3000× *g* rpm, 30 min) Dialysis (3500 Da, 5 days)	3.5	n/a	[125]
Gene therapy Bioimaging	BT-474 cells	HER3 siRNA	HER3	Hydrothermal	Citric acid monohydrate Ethylenediamine + Branched Polyetherimide	200 °C, 4 h	Branched PEI -functionalized N-doping	Centrifugation (13,000× *g* rpm, 20 min) Dialysis (1000 Da, 96 h)	5.5	n/a	[126]
Bone regeneration promotion	Pre-osteoblast cells	n/a	n/a	Microwave-assisted	Ascorbic acid PEI	450 W, 5 min	Polymer passivation	Centrifugation (12,000× *g* rpm, 15 min) Filtration with filter membrane (0.22 μm)	2–3	n/a	[127]
Modulation of amyloid beta fibrillation and toxicity Bioimaging	SH-SHSY5Y cells	n/a	Aβ42	Hydrothermal	L-lysine D-lysine	170 °C, 2 h	Chirality	Dialysis (2000 Da, 24 h)	3.9	n/a	[128]
Photosynthesis enhancement (growth stimulation)	Chloroplasts (in vivo and in vitro)	n/a	n/a	Microwave-assisted	Glutathione Formamide	800 W, 3 min	N, S-doping	Centrifugation (10,000× *g* rpm, 5 min) Dialysis (3500 Da, time n/a)	3.8	18.5	[40]
Gene therapy Bioimaging	A549 cells	n/a	n/a	Solvothermal	PEI 600 Da	180 °C, 5 h	functionalization with hydrophobic chains with different degrees of substitution	Filtration with filter membrane (0.45 μm) Dialysis (1000 Da, 3 days)	6.4	PEI-CD: 21.49	[129]
Gene therapy Bioimaging	Kaposi’s sarcoma-associated herpes virus (KSHV) cells (in vivo and in vitro)	locked nucleic acid (LNA)	Viral miRNAs	Microwave-assisted	Citric acid PEI	1150 W, 3 min	locked nucleic acid (LNA) functionalized	Centrifugation (13,000× *g* rpm, 10 min) Dialysis (14,000 Da, 2 days)	3.0	n/a	[130]
Bioimaging	*Fusarium oxysporum*, *S. aureus*, *P. aeruginosa*, *B. subtilis*, *E. coli*, MCF-7 and HepG2 cells	n/a	n/a	Microwave-assisted	Pomegranate Watermelon extract PEG 200	Microwave radiation, 2 min at the interval of 10 s each	Polymer passivation	n/a	1–5	n/a	[131]
Bioimaging	HeLa cells	Human serum albumin	gp60	Pyrolysis	Tartaric acid L-tyrosine	220 °C, 30 min	HSA modified	n/a	5.50	n/a	[85]
Drug delivery Bioimaging	HeLa and HEK 293 T cells	Doxorubicin	Folate receptors	Hydrothermal	Citric acid BPEI folic acid	200 °C, 4 h	Polymer passivation β-cyclodextrin modified	Centrifugation (10,000× *g* rpm, 15 min) Dialysis (100–500 Da, 48 h)	2.8	64.5	[87]
Antioxidant capacity enhancement	Arabidopsis thaliana seedlings	Phenol	Horseradish peroxidase	Electrochemical	Ammonia	certain voltage with graphite electrodes; electrolyte of a certain concentration of ammonia aqueous solution; several days	N-doping Horseradish peroxidase modified (immobilization)	Centrifugation (10,000× *g* rpm, 15 min) Dialysis (3500 Da membrane)	n/a	n/a	[88]
Gene therapy Bioimaging	HeLa cells	Cy5-labelled DNA	n/a	Hydrothermal	Citric acid Pcyclen	180 °C, 5 h	Polymer passivation	Centrifugation (12,000× *g* rpm, 10 min) Dialysis (1000 Da, 1 day)	1.8	3.25	[89]
					Citric acid Ptaea				5.4	1.73	
Antioxidant capacity	human dermal fibroblasts	n/a	n/a	Microwave-assisted	L-lysine Propylene carbonate	240 °C, 120 min	Polymer passivation	Dialysis (500–1000 Da, time n/a)	2–5	23.3	[90]
Drug delivery Bioimaging	MCF-7 cells	Methotrexate	Folate receptors	Microwave-assisted	Gelatin PEG	600 W, 10 min	Polymer passivation	Centrifugation (12,000× *g* rpm, 15 min)	6	34	[91]
Bioimaging: Microorganism imaging and cancer/normal cells differentiation In vivo nude mice bearing U14 tumours diagnosis	Hep G2, A549, L02 and U14 and AT II cells *Staphylococcus aureus*, *Escherichia coli*, *Trichoderma reesei,* and *Saccharomyces cerevisiae* cells Zebrafish	n/a	Fe^3+^/Glutathione	Solvothermal	N-[3-(Trimethoxysilyl)propyl]ethylenediamine (DAMO) glycerol	degassing process with nitrogen, 2 min 260 °C, 12 h	n/a	Centrifugation (8000× *g* rpm, 10 min) Dialysis (~1 kDa, 2 days)	~6.1	45	[132]
Bioimaging Drug delivery	Nucleolus	Protoporphyrin IX (PpIX)	RNA (nucleolus) caveolae-mediated endocytosis (CvME), lipid raft-mediated endocytosis (LrME), clathrin-mediated endocytosis (CME), and macropinocytosis (cellular uptake)	Hydrothermal	*m*-phenylenediamine *L_-_*cysteine	160 °C, 10 h	dicyclohexylcarbodiimide (DCC) 1-hydroxybenzotriazole (HOBt)	Centrifugation (15,000× *g* rpm, 15 min) Dialysis (1000 Da, 2 days)	CQs: 3.8	~8.4	[133]
									CDs-PpIX: 25.2	62.1	
Bioimaging in vivo	Caenorhabditis elegans (C. elegans) BALB/c mice	n/a	n/a	(i) Hydrothermal treatment (Blue-CQDs) (ii) ortho-phosphoric acid treatments (Green-CQDs)	beetroot extract (*Beta vulgaris*)	150 °C, 16 h	n/a	Centrifugation (1500× *g* rpm, 2500 rpm and 4000 rpm, 10 min each)	(i) 5 (ii) 8	(i) 6 (ii) 5	[134]
						100 °C, 2 h					
Gene delivery	HEK 293 T, NIH 3T3, COS-7, and HepG2 cells B16F10 and A549 cells Primary 3T3-L1 and mESCs cells	pDNA	Cytomembrane, Karyotheca, endosome/lysosome, membranes	Solvothermal	tetrafluoroterephthalic acid (fluorine) terephthalic acid 1.8 kDa branched-polyethyleneimine (1.8 k b-PEI) 25 kDa branched-polyethyleneimine (25 k PEI)	180 °C, 6 h	fluorine-doped cationic CDs (FCDs): tetrafluoroterephthalic acid (fluorine) pDNA	Dialyzed (3.5 kDa, time n/a)	4.8 (FCDs) 150–200 (FCDs/DNA)	n/a	[135]
Drug delivery	Porphyromonas gingivalis (P. gingivalis)	metronidazole (MET)	n/a	Hydrothermal	chlorophyll	240 °C, 18 h	n/a	Centrifugation (5000× *g*, 30 min) Filtration (0.22 mm) Ultrafiltration membranes (3 kDa and 30 kDa)	2–4	56%	[136]
Cancer detection	-	-	-	Hydrothermal (CDs)	sweet lemon peel	180 °C, 3 h (CDs)	1-(3- Dimethylaminopropyl)-3-ethyl carbodiimide (EDC)	Filtration Concentration rotary evaporator	1.5 –6.5	n/a	[137]
	Triple negative breast cancer (TNBC)	polyamidoamine (PAMAM) dendrimers (G1, G2 and G3)	Copper (II) overexpressed in TNBC MDA-MB-231 cells	Hydrothermal (CD-PAMAM (CDP): CDP1, CDP2, CDP3	CDs	RT, 16 h	N-hydroxysuccinimide (NHS) ethylenediamine (EDA) PAMAM dendrimers G1, G2 and G3	Dialysis (3.5 kDa, 3 days)	n/a		
Gene therapy Bioimaging agent for TNBC cells	Triple negative breast cancer (TNBC)—MDA-MB 231 cell	eGFP-pDNA	RGD receptor αvβ3 integrin	Hydrothermal (RGDS decorated CDP3 (CDP3-RGDS))	CDP conjugated (PAMAM) dendrimers G3 (CDP3)	RT, 16 h	EDC and NHS RGDS peptide	Dialysis (3.5 kDa, 3 days)	7–27	n/a	
Skin permeability	methicillin-sensitive Staphylococcus aureus (ATCC 6538) methicillin-resistant Staphylococcus aureus (ATCC 4300) Escherichia coli (ATCC 25922) Staphylococcus epidermidis (ATCC 49134) Bacillus cereus (ATCC 14579)	n/a	n/a	Hydrothermal	polyethyleneimine (PEI) citric acid (CA)	250 °C, 4 h	N-doping	Dialysis (≥12,000 Da, 24 h)	70–10	PEI:CA ratio (1:0,5)–31 (1:1)–53 (1:2)–7.6	[138]
Induction of potent humoral and cellular immune responses	LnCaP cells Bone marrow-derived dendritic cells (BMDCs) Mouse leukemic monocyte macrophage cells (RAW264.7) BALB/c mice OVA-specific CD4 +T and CD8 +cytotoxic T lymphocytes (CTLs)	ovalbumin (OVA) antigen	antiOVA IgG1, IgG2a and IgG2b	Hydrothermal	N,N′-dodecyl2-hydroxyl-N,N,N′,N′-tetramethyl diammonium dichloride (BQAS)	180 °C, 12 h	ovalbumin (OVA)	Centrifugation (10,000× *g* rpm,15 min) Dialysis (500 Da, 12/12 h)	3.74	7.8	[139]
Cancer bioimaging	27 cancer cell lines of different origin, a side population (SP) of cancer stem-like cells isolated from MDA-MB-231 cells27, brain cancer stem cell lines (BCSCs), 12 patient-derived and 18 non-cancerous cell lines mice bearing HeLa tumours	n/a	large neutral amino acid transporter (LAT1)	Hydrothermal	LAAM TC-CQDs: 1,4,5,8-tetraminoanthraquinone (TAAQ) citric acid (CA)	180 °C, 2 h	n/a	Silica column chromatography using mixtures of dichloromethane and methanol (10:1) as eluents for three rounds	2.45	6.8 (in water)	[140]
Drug delivery	cancer cells and non-cancerous cells	DNA-damaging chemotherapy drugs: topotecan hydrochloride (TPTC), DOX and hydroxycamptothecin	Nuclei DNA				topotecan hydrochloride (TPTC) DOX hydroxycamptothecin	Dialysis (MWCO n/a, time n/a)			
Brain cancer imaging and treatment	mice bearing U87 brain tumours	topotecan hydrochloride (TPTC)	LAT1 in Blood-Brain Barrier (BBB)								
Tumour-specific imaging and drug delivery	HeLa and CCC-ESF-1 cells	1,4-diaminoanthraquinone (1,4-DAAQ	LAT1-mediated tumour-specific		1,4-CQDs: 1,4-diaminoanthraquinone (1,4-DAAQ) citric acid (CA)		n/a	Silica column chromatography using mixtures of dichloromethane and methanol (20:1) as eluents	n/a	n/a	
		1,5-diaminoanthraquinone (1,5-DAAQ)			1,5-CQDs: 1,5-diaminoanthraquinone (1,5-DAAQ) citric acid (CA)				n/a	n/a	
		2,6-diaminoanthraquinone (2,6-DAAQ)			2,6-CQDs: 2,6-diaminoanthraquinone (2,6-DAAQ) citric acid (CA)				n/a	n/a	
		n/a		Solvothermal	Phe-CQDs: phenylalanine (Phe)	180 °C in oven, 8 h	n/a	Silica column chromatography using mixtures of dichloromethane and methanol (50:1) as eluents	n/a	n/a	
Bioimaging	HeLa cells BALB/c mice	ibuprofen	n/a	Microwave- assisted	Triethanolamine Ibuprofen	700 W, 8 min	N-doping	Centrifugation (8000× *g* rpm, 10 min) Dialysis (14 kDa, 3 days)	6.99	22	[141]
Anti-inflammatory	BALB/c mice		COX-1 or COX-2								
Bioimaging	Mung beans (Vigna radiata) Human MG-63 osteosarcoma cell line Laboratory-bred strain golden hamsters	n/a	Malachite green	Hydrothermal	Sandalwood powder	200 °C, 8 h	n/a	Centrifugation (15,000× *g* rpm, 20 min) Filtration (0.22 µm)	3.5	12	[142]
Bioimaging	Hepg2 cells	n/a	n/a	Hydrothermal	Citric acid borax *p*-phenylenediamine	180 °C, 5 h	N, B-doping	Filtration (0.22 μm) Dialysis (500 Da, 24 h)	3.53	n/a	[143]

n/a: not available. RT: Room Temperature.

**Table 4:** Summarized examples of carbon quantum dots used fo0r chemical sensing.

Analyte(s)	Sensing Mechanism	CQDs Synthesis Method	Precursors	Reaction Conditions	Modifications and Functionalization	Purification	Average Size (nm)	QY (%)	Linear Range	Limit of Detection	Ref.
Folic acid	dynamic quenching	Hydrothermal	Aconitic acid (AA) 1,2-ethylenediamine (EDA)	150 °C, 5 h	N-doping	n/a	1.6	56.5	1–100 µm	40 nm	[31]
Fe (II)	static quenching	Hydrothermal	succinic acid glycerol	250 °C, 6 h	n/a	Dialysis (1000 Da, 3 days) Centrifugation (10,000× *g* rpm, 20 min)	2.3	11	10–50 μm	21.9 μm 0.7 μm	[33]
H_2_O_2_							4.6		100–500 nm		
Picric acid	FRET	Hydrothermal	L-Lysine thiourea	220 °C, 5 h	N,S-doping	Centrifugation (10,000× *g* rpm, 10 min)	6.86	53.19	1–10 μm	0.24 μm	[34]
Cr (VI)	IFE	Continuous hydrothermal flow	citric acid ammonia	450 °C	N-doping	Filtration (0.22 µm alumina membrane) Dialysis (30 kDa, 1 kDa, time n/a)	3.3 ± 0.7	14.91 ± 0.24	5–250 ppm	0.365 ppm	[22]
Tetracycline	static quenching	Microwave-assisted	glycerol urea	700 W, 4 min	N-doping	Centrifugation (3000× *g* rpm, 4 min) Dialysis (12,000 Da, 3 days)	13.2	9.8	0.50–25 μm	165 nm	[36]
Levofloxacin	ET	Microwave-assisted	L-cysteine ammonium citrate	750 W, 2.5 min	N,S-doping	n/a	2	64	0.01–70 mg L^−1^	5.1 μg L^−1^	[24]
Alkaline phosphatase	fluorescence quenching (ON-OFF)	Electrochemical	Pyrocatechol ethanediamine	10 V DC, 30 min	N-doping	Dialysis (3500 Da, 1 day)	3.15	30.6 ± 0.12	5–500μm	0.5 μm	[42]
Alkaline phosphatase	ratiometric fluorescence	Electrochemical	[BMIM][BF4]	15 V DC, 4 h	Ionic liquid-functionalization	Centrifugation (10,000× *g* rpm, 20 min) Dialysis (1000 Da, 2 days)	2.75	23.4	0.04–3.2 U L^−1^ (12 to 960 pm)	0.012 U L^−1^ (3.6 pm)	[43]
Fe (II)	fluorescence quenching and ratiometric fluorescence	Ultrasonic	dopamine hydrochloride dimethylformamide	pulsed mode (600 W, 20 kHz) with a frequency of 1 s, 8 h	N-doping	Centrifugation (n/a rpm, time n/a) Dialysis (500 Da, time n/a)	2.8–4.1	3.6	0–50 μm	38 nm	[46]
Fe (III)	fluorescence quenching	Pyrolysis	Aloe-Vera extract	190 °C, 20 min	n/a	Dialysis (300 Da, time n/a)	6–8	12.3	70 ppb–10 ppm	33 ppb	[51]
Dopamine	ET	Hydrothermal	polyacrylamide	180 °C, 5 h	N-doping	Centrifugation (13,000× *g* rpm, 20 min) Filtration (0.22 µm)	3	23.1	0.1–200 μm	0.05 μm	[56]
Cr (VI)	IFE	Hydrothermal	*p*-phenylenediamine ammonia	200 °C, 8 h	N-doping	Dialysis (500–1000 Da, 3 days)	3.2	13.2	0.0375−3 μm	22.5 nm	[57]
2,4,6-Trinitrophenol (TNP)									19−27 μm	3.69 μm	
ascorbic acid									5–50 μm	3.2 μm	
Fe (III)	dynamic quenching	Hydrothermal	*o*-phenylenediamine (OPD) 2,5 pyridinedicarboxylic acid (2,5 PDC)	180 °C, 5 h	N-doping	Centrifugation (n/a rpm, time n/a) Filtration (0.22 µm) Dialysis (3500 Da, 1 day)	4.7	47	3–60 μm	0.31 μm	[58]
Cu (II)									0.5–15 μm	56 nm	
Amoxicillin	fluorescence enhancement	Hydrothermal	citric acid boric acid	210 °C, 24 h	B-doping	Filtration (0.22 µm)	2.3	30.85	1.43–429.12 μm	0.825 μm	[60]
Potassium sorbate	FRET	Hydrothermal	phenylboronic acid	200 °C, 10 h	B-doping	Centrifugation (7060× *g*, 15 min) Filtration (0.22 µm) Dialysis (100 Da, 48 h)	3.3	12	0.20–24 μm	6.1 nm	[61]
Vitamin B12									0.20–30 μm	8.0 nm	
Cd (II)	fluorescence enhancement	Hydrothermal	*o*-phosphorylethanolamine citric acid	180 °C, 12 h	N,P-doping	Dialysis (1000 Da, overnight) Filtration (0.22 µm)	1.6	8.17	0.5–12.5 μm	0.16 μm	[63]
Cu (II)	IFE	Hydrothermal	Glucose concentrated H_3_PO_4_ polyethylene glycol diamine	90–100 °C, 9 h	N,P-doping	Centrifugation (14,000× *g* rpm, 30 min) Dialysis (1000 Da, 1 day)	3.5	25	4×10^−9^–4 × 10^−7^ M	1.5 nm	[64]
Curcumin	IFE	Self-exothermic reaction	Glucose 1,2-ethylenediamine concentrated phosphoric acid	acid-base neutralization spontaneous heat,6 min	N,P-doping	Dialysis (1000 Da, 3 days)	3.5 ± 0.2	9.59	0.5–20 µm	58 nm	[65]
MnO_4_^-^	fluorescence quenching	Microwave-assisted	1,6-hexanediamine dihydrochloride dimethyl sulfoxide	180 °C, 35 min	N,S-doping	Filtration (0.22 µm) Dialysis (500 Da, 36 h)	4.35	24	1–20 μm	0.34 μm	[69]
Cr_2_O_7_^2-^									Linearity not satisfied	0.23 μm	
Permanganate	static quenching	Hydrothermal	p-amino salicylic acid boric acid ethylene glycol dimethacrylate	180 °C, 5 h	N,B-doping	Centrifugation (12,000× *g* rpm, 15 min) Filtration (0.22 µm) Dialysis (1000 Da, 1 day)	5	19.6	0.05–60 μm	13 nm	[71]
Captopril									0.1–60 μm	0.03 μm	
Glucose	aggregation-induced emission (AIE)	Hydrothermal	4-carboxyphenylboronic acid *o*-phenylenediamine rhodamine B	150 °C, 5 h	N,B-doping	Centrifugation (rpm n/a, time n/a)	30	46	32 μm–2 mm	8 μm	[73]
Tetracycline	IFE	Ultrasonic	thiamine pyrophosphate	alkaline solution, room temperature, 240 min	N,S,P-doping	Dialysis (2000 Da, 48 h)	3.19	20.5	0.1−20 μm	0.0444 μm	[74]
Cr (VI)	IFE	Hydrothermal	p-aminobenzenesulfonic acid tetrakis(hydroxymethyl)phosphonium chloride	180 °C, 10 h	N,S,P-doping	Filtration (0.22 µm) Dialysis (500 Da, 72 h)	2.18	36.8	1–500 μm	0.23 μm	[75]
miRNA-9	fluorescence enhancement	Hydrothermal	Thiourea *o*-phenylenediamine	200 °C, 8 h	N-doping	Filtration (0.22 µm) Dialysis (MWCO n/s, time n/a)	3.6 ± 0.5	n/a	4.00–250 fm	0.57 ± 0.12 fm	[144]
Thiourea	static quenching (ON-OFF)	Hydrothermal	ammonium citrate dextrin	165 °C, 5.5 h	N-doping	Filtration (0.22 µm)	1	17	0.90–10.0 μm	0.15 μm	[145]
Catechol	photoelectron transfer	Hydrothermal	citric acid sodium tetraphenylborate	180 °C, 8 h	B-doping	Extraction method with CCl_4_/H_2_O	8	42	1–50 nm	0.25 nm	[146]
Glutathione									2–100 nm	0.5 nm	
*L*-cysteine	Chemiluminescence enhancement	Hydrothermal	Starch	190 °C, 2 h	n/a	Centrifugation (15,000× *g* rpm, 20 min)	3.2	n/a	10.0–100 μm	8.8 μm	[147]
Dopamine	fluorescence quenching	Hydrothermal	anhydrous citric acid ethylenediamine	150 °C, 2 h	N-doping	Dialysis (3000 Da, 2 days)	n/a	n/a	0.05–15 μm	0.035 μm	[148]
α-lipoic acid									0.5−55 μm	0.39 μm	
Ca (II)	static quenching	Hydrothermal	1st) citric acid and ethylenediamine 2nd) thylenebis(oxyethylenenitrilo)tetraacetic acid (EGTA)	180 °C, 8 h + 150 °C, 4 h	EGTA functionalized	Centrifugation (10,000× *g* rpm, time n/a) Dialysis (500 Da, 1 day)	5	n/a	15−300 μm	0.387 μm	[149]
Fe (II)	fluorescence quenching	Pyrolysis	mango (*Mangifera indica*) leaves	300 °C, 3 h	n/a	Vacuum filtration	2 to 10	18.2	0–1000 μL of 10 ppm Fe (II)	0.62 ppm	[150]
Cu (II)	static quenching	Pyrolysis	*Vitis vinifera* grape juice	200°C, 6 h	Polyamine functionalized	Filtration (0.45 µm)	8	32.1	0.07–60 μm	0.02 μm	[151]
S^2-^									0.8−95 μm	0.24 μm	
Cr (VI)	dynamic and static quenching	Hydrothermal	Potato	150 °C, 90 min 200 °C, 2.5 h	n/a	Filtration Paper filtration (11 µm) Centrifugation (12,000× *g* rpm, 10 min)	5.97	6.08	0.5−100 μm	0.012 μm	[152]
Fe (III)									0.5−5 μm	0.000549 μm	
Pb (II)	ET	Hydrothermal	L-lysine L-glutathione	190 °C, 24 h	Thiol-functionalized	Filtration Centrifugation (8000× *g* rpm, 20 min)	5	n/a	0−20 μm	2.2 μm	[83]
Glucose	fluorescence quenching	Microwave-assisted	citric acid tyramine	800 W, 120 s	N-doping; Tyramine hybridized	Centrifugation (9000× *g* rpm, 15 min) Filtration (0.45 µm)	17.5	11.0 ± 0.8	10^−6^–10^−5^ M	n/a	[84]
Pb (II)	static quenching	Microwave-assisted	Urea citric acid boric acid	700 W, 4 min	N,B-doping; BSA modified	Dialysis (500 Da membrane, 1 day)	<5	n/a	1−10 ppb	0.08 ppb	[86]
Fe (III)	fluorescence static quenching	Solvothermal	Glycerol N-[3-(trimethoxysilyl)propyl]ethylenediamine (DAMO)	degassing process with nitrogen, 2 min 260 °C, 12 h	Silicon and Nitrogen co-doping	Centrifugation (8000× *g* rpm, 10 min) Dialysis (~1 kDa, 2 days)	~6.1	45	0.1–100 µm	16 nm	[132]
Fe^3+^	Fluorescent quenching	Hydrothermal	Thiamine hydrochloride (Vitamin B1) ethylenediamine	200 °C, 12 h	S-doping	Centrifugation (rpm n/a, time n/a) Dialysis (3500 Da, 3 days)	3.2	4.4	0.1−1.0 mm	177 nm	[153]
tetracycline	Fluorescent static quenching	Microwave-assisted	Xylan NH_4_OH	200 °C, 200 W, 10 min	N-doping	Filtration (0.22 μm)	7.89	4	0.05–20 μm	6.49 nm	[154]
					Xylan-derived N-CQDs without N-doping			2			
Hematin	Fluorescent static quenching (IFE)	Solvothermal	p-aminobenzoic acid (PABA)	180 °C, 12 h	n/a	Silica column chromatography	11.8	n/a	0.5–10 μm	0.25 μm	[155]
glutathione	CQDs-H_2_O_2_- TMB (3, 3′, 5, 5′-tetramethylbenzidine) system	Acid refux	wood soot HNO_3_	140 °C, 12 h	n/a	Dialysis (MWCO n/a, 2 days) Centrifugation (16,000× *g* rpm, 15 min) Ultra-filtration	2.3	n/a	0.05–20 μm	0.016 μm	[156]
Pesticides: propanyl parathion dimethoate chlorpyrifos pyrimicarb	FRET	Hydrothermal	riboflavin	160 °C (1, 2, 5 h) 180 °C (1, 2, 5 h) 200 °C (1, 2, 5 h)	n/a	Dialysis (12 h/12 h) of “CD6 (180 °C, 5 h)”	3.47 ± 0.02 (CD6)	n/a	n/a	n/a	[157]
H_2_O_2_	M-CQDs-H_2_O_2-_TMB (3, 3′, 5, 5′-tetramethylbenzidine) system	Hydrothermal	Mustard seeds	180 °C, 4 h	n/a	Centrifugation (15,000× *g* rpm, 20 min)	4.58 ± 0.26	4.6	0.02−0.20 mm	0.015 mm	[158]
ascorbic acid									10−70 µm	3.26 µm	
Acetone	enhancement in PL emission	Hydrothermal	citric acid borax *p*-phenylenediamine	180 °C, 5 h	N, B-doping	Filtration (0.22 μm) Dialysis (500 Da, 24 h)	3.53	n/a	1–200 μm	0.54 μm	[143]
Dopamine	static quenching mechanism								0.1–70 μm	11 nm	
m-benzenediol (resorcinol)	Fluorescence enhancing	Hydrothermal	*Cryptococcus*	160 ℃, 1 h	n/a	Centrifugation (5000× *g* rpm, 5 min) Filtration (0.22 mm) Dialysis (100 Da, 12 h)	4–9	14.13	2 × 10^−8^–4 × 10^−4^ m	8.68 nm	[159]
p-benzenediol ((hydroquinone)	Fluorescence quenching								4 × 10^−9^–2 × 10^−5^ M	6.7 nm	
Cu (II)	Fluorescence quenching	Dry-Pyrolysis	Finger millet ragi (*Eleusine coracana*)	300 °C (step of 5 °C/min), 3 h at 300 °C	n/a	Filtration (0.22 µm)	6	n/a	0–100 μm	10 nm	[160]
Ellagic acid	Fluorescent static quenching (inner filter)	Hydrothermal	citric acid diethylenetriamine	180 °C, 10 h	N-doping	Dialysis tube (500 Da, time n/a)	3.16	84.79	0.01–50 μm	0.01 μm	[161]
Dimethoate	fluorescence static quenching	Dry-Pyrolysis	pork rib bones from food waste	1st) 700 °C, 5 h 2nd) Centrifugation (6000× *g* rpm, 6 min) 3rd) 200 °C, 10 h	dithizone	Filtration (0.22 μm)	4.2 ± 1.2	n/a	0.15 μm–5.0 μm	0.064 μm	[162]
curcumin in dietary foods	IFE and dynamic interaction	Solvothermal	*m*-phenylenediamine citric acid	180 °C, 12 h	N-doping	Dialysis (500–1000 Da, 24/24/24 h)	4.5	61.7	0.01–25.0 μm	28.7 nm	[163]
Diazinon	Fluorescent static quenching	Hydrothermal (Aqueous extracts)	Blue rose petals	200 °C, 2 h	n/a	Filtration	37	46	0.02–10 µm	0.01 µm	[164]
			Red rose petals				39	44			
			Yellow rose petals				33	48			
		Solvothermal (Alcoholic extracts)	Blue rose petals				30	43			
			Red rose petals				27	46			
			Yellow rose petals				26	47			
Ovalbumin (OVA)	C-MIPs@FITC	Hydrothermal	citric acid ethylenediamine	200 °C, 5 h.	N-doping	Dialysis (MWCO n/a, 24 h)	n/a	n/a	0.05–2 μm	15.4 nm	[165]
			SiO_2_ NPs (i) OVA (i) APTES (i) TEOS (ii) NH_3_.H_2_O (ii) FITC (iii)	(i) Stirring 1 h (ii) Stirring 24 h (iii) Stirring 2 h	(MIPs@FITC) OVA fluorescent imprinted nanoparticles	(ii) centrifugation and washing w/methanol/acetic acid (9:1, *v*/*v*) (iii) washing w/ethanol	90				
			CDs MIPs@FITC PBS	mixing	(C-MIPs@FITC) molecularly imprinted ratiometric fluorescence nanosensor	n/a	n/a				

n/a: not available; IFE: Inner Filter Effect; ET: Electron Transfer; BSA: Bovine Serum Albumine; PL: Photoluminescence.

**Table 5:** (**A**) summarized examples of carbon quantum dots used for optoelectronics—LEDs. (**B**) Summarized examples of carbon quantum dots used for optoelectronics–Solar converters.

(A)											
Application	CCT (K)	Lmax (cd m−2)	ηc (cd A−1)	CQDs Synthesis Method	Precursors	Reaction Conditions	Modifications and Functionalization	Purification	Average Size (nm)	QY (%)	Ref.
LEDs	n/a	1882–4762	1.22–5.11	Solvothermal	Phloroglucinol (PG)	200 °C, 2 h, 5 h, 9 h, and 24 h	n/a	Silica gel column chromatography (Dichloromethane and Methanol (variable)	1.9–3.9	blue CQDs: 66 green CQDs: 72 yellow CQDs: 62 red CQDs: 54	[166]
LEDs	6000	n/a	n/a	Reflux	Diammonium hydrogen citrate Urea	180 °C, 15 min	N-doping	Centrifugation (6000× *g* rpm, 20 min)	4–10	16	[167]
LEDs	n/a	2–174	9 × 10^−4^–2 × 10^−3^	Hydrothermal or Hot injection	Citric acid HDA/urea/EDA/PDA	200 °C, 5 h or 160 °C, 16 h	N-doping HDA/EDA/PDA hybridization	Centrifugation (4500× *g* rpm, Time n./a)	3.6, 5.4, 3.3, 31.7	EDA-CQDs: 43 PDA-CQDs: 8 HAD-CQDs: 40 Urea-CQDs: 35	[168]
LEDs	2520	n/a	n/a	Hydrothermal	2,7-dihydroxynaphthalene Ethylenediamine	180 °C, 12 h	N-doping	Centrifugation (8000× *g* rpm, 5 min)	3.31	62.98	[169]
LEDs	n/a	n/a	n/a	Microwave-assisted	Citric acid *o*-Phenylene-diamine (oPD)	125 °C, 5 min	N-doping	Dialysis (1000 Da, 30 min)	1.1	5.4	[38]
LEDs	6250	n/a	n/a	Reflux	Citric acid Tris-HMA	225 °C, 20 min	N-doping Polymer passivation	Dialysis (1000 Da, 2 days)	3.35	23	[92]
LEDs	3913, 5994, 10000	n/a	n/a	Solvothermal	*o*-Phenylenediamine 4-aminobenzenesulfonic acid folic acid boric acid acetic acid terephthalic acid tartaric acid	180 °C, 12 h	N-doping	Dialysis (MWCO n/a, Time n/a) in ethanol	3.01	b-CQDs: 25 c-CQDs: 36 g-CQDs: 28 yg-CQDs: 72 y-CQDs: 45 o-CQDs: 59 r-CQDs: 47 dr-CQDs: 52 w-CQDs: 39	[170]
**(B)**											
**Application**	**Jsc (mA/cm2)**	**Voc (V)**	**η (%)**	**CQDs Synthesis Method**	**Precursors**	**Reaction Conditions**	**Modifications and Functionalization**	**Purification**	**Average Size (nm)**	**QY (%)**	**Ref.**
Solar converters	22.2	1.05	15	Hydrothermal	Citric acid Urea	200 °C, 6 h	TiO_2_	Wash with deionized water and ethanol numerous times	30–40	n/a	[171]
Solar converters	n/a	n/a	n/a	Hydrothermal	Citric acid Ethylenediamine TRIS	200 °C, 6 h	N-doping	Dialysis (3000 Da, 2 h)	2	50	[172]
				Hot-injection	PbBr_2_/PbCl_2_ salts	-	n/a	-	11–14	CsPb (Br0.8Cl0.2)3 QDs: 70 CsPb(Br0.2I0.8)3 QDs: 60	
Solar converters	12.67–18.49	0.46–0.48	3.26–4.97	Microwave-assisted	Citric acid Urea	800 W, 5 min	N-doping	Filtration with a cylinder filtration membrane filter (0.22 μm)	2.9	80	[173]
Solar converters	35.98	0.62	17.86	Hydrothermal	Citric acid KH-792 Thiourea	180 °C, 12 h	N-doping	Wash with hexane, 3 times Dialysis (MWCO n/a, 24 h)	2–3	20.68	[174]
Solar converters	17.4	0.738	9.04	Hydrothermal	Lotus root powder Sulphuric acid Ammonium hydroxide	170 °C, 6 h	S- or N-doping	Dialysis (3500 Da, Time n/a)	4–5	n/a	[175]
Solar converters	1.60	0.54	1.20	Solvothermal	Jaggery syrup Urea	140 °C, 2.5 h	N-doping	Centrifugation (12,000× *g* rpm, 30 min)	9.5	9.8	[176]
Solar converters	4.5	0.495	1.6	Hydrothermal	Phloroglucinol	200 °C, 9 h + 70 °C, 30 min	n/a	Dialysis (1000 Da, 12 h)	1.74	40	[177]

n/a: not available.

**Table 6:** Summarized examples of carbon quantum dots used for photocatalysis.

Target/ Contaminant	Photocatalysis Mechanism	CQDs Synthesis Method	Modifications and Functionalization	Precursors	Reaction Conditions	Purification	QY (%)	Average Size (nm)	Degradation (%)	Degradation Time (min)	Ref.
Rhodamine B Methylene blue	Oxidation by formation of holes and superoxide radical anions	Microwave assisted	N-doping	Citric acid 1,2-Phenylenediamine	700 W, 7 min	Filtration and SEC	39.2	2	70	150	[114]
Acid Blue	Formation of reactive oxide species (ROS)	Hydrothermal	N-doping	Grass	180 °C, 2 h	Wash with distilled water	n/a	<10	100 (for all)	30	[178]
Acid Red										30	
Eosin Y										90	
Eriochrome Black T										Immediate decomposition no radiation required	
Methyl orange										Immediate decomposition no radiation required	
Methylene blue										90	
Rhodamine B Methylene Blue	Oxidation by formation of holes and active oxygen/hydroxyl radicals	Hydrothermal	n/a	Palm powder	200 °C, 7 h	Filtration with membrane filter (0.22 μm) Dialysis (500 Da membrane, 48 h)	0.9	3.54	71.7 and 94.2, respectively	45 (both)	[179]
Methyl viologen	photoinduced electron transfer	Hydrothermal (for amorphous synthesis (a-))	n/a	Citric acid (Cit)	180 °C, 24 h	-	1.0 a-Cit-CDs	9–12	3.45 × 10^−8^ M s^−1^	n/a	[180]
				Glucose (Glu)	200 °C, 24 h	Centrifugation (n/a time) and Filtration	1.8 a-Glu-CDs		0.65 × 10^−8^ M s^−1^		
				Fructose (Fru)	200 °C, 24 h		0.3 a-Fru-CDs		4.37 × 10^−8^ M s^−1^		
		Pyrolytic (for graphitic synthesis (g-))		Citric acid (Cit)	220 °C, 48 h	Dialysis (1000 Da membrane, 24 h)	1.2 g-Cit-CDs	7–9	5.06 × 10^−8^ M s^−1^		
				Glucose (Glu)	220 °C, 48 h		2.3 g-Glu-CDs	2–7	1.07 × 10^−8^ M s^−1^		
				Fructose (Fru)	220 °C, 48 h		0.7 g-Fru-CDs	0.5 μm–1.5 μm	0.67 × 10^−8^ M s^−1^		
Tetracycline Bisphenol A Rhodamine B	Direct hole oxidation reaction	Hydrothermal	n/a	n/a	n/a	n/a	80	5	60 73 91.8	120 150 20	[181]
Rose bengal	Formation of singlet oxygen	Bottom-up condensation	P-doping	Pluronic F-68	n/a	n/a	31	<21	86	180	[182]
CO_2_	Reduction by accumulated electrons under the assistance of H^+^	Microwave-assisted	N, S-doping	Citric acid Thiourea	800 W, 7 min	Dialysis (MWCO n/a, 24 h)	n/a	3.5	n/a	360	[183]
Tetracycline	Oxidation by formation of holes and superoxide radical anions	Hydrothermal	N-doping	Ammonium citrate Ethylenediamine	200 °C, 5 h	Dialysis (1000 Da, 24 h)	n/a	8	97	25	[184]
Crystal violet dye	Oxidation by formation of holes and photo-generated electrons	Hydrothermal and mixed-calcination	n/a	Ascorbic acid Glycol	160 °C, 70 min	n/a	n/a	4–9	70	300	[185]
Amoxicillin	Oxidation by formation of holes and hydroxyl radicals	Hydrothermal and calcination	potassium titanate (K_2_Ti_6_O_13_)	n/a	n/a	n/a	80	n/a	100	90	[186]
Sulfamethazine	Oxidation by formation of holes, superoxide radical anions and photo-generated electrons	Calcination	N-doping	Citric acid Urea	550 °C, 3 h	n/a	n/a	2–5	97.3	50	[187]
Indigo carmine	Oxidation by formation of holes and photo-generated electrons	Solvothermal	N-doping	Aniline	180 °C, 10 h	Centrifugation (10 000× *g* rpm, 5 min) Dialysis (3500 Da, 24 h)	0.25	2.0	97	120	[188]
				2-aminonaphthalene			0.11	2.2			
				2-anthracylamine			0.28	2.6			
				1-aminopyrene			0.14	2.2			
Benzene *p*-xylene Toluene	CQDs/TiO_2_ nanocomposites improved interfacial charge transfer; increased light absorption; narrower bandgap	Pyrolysis	CQDs/TiO_2_ nanocomposites	Citric acid	180 ℃, 40 h	n/a	n/a	2.4	31 64 99	140	[189]

n/a: not available; SEC: size-exclusion chromatography.

**Table 7:** Summarized examples of carbon quantum dots used as antimicrobials and antivirals.

Application	Target	Targeting Ligands	Receptor/Target	CQDs Synthesis Method	Precursors	Reaction Conditions	Modifications and Functionalization	Purification	Average Size (nm)	QY (%)	Ref.
Antimicrobial	*Escherichia coli* (*E. coli*) *Staphylococcus aureus* (*S. aureus*)	n/a	n/a	Pyrolysis	Aloe-Vera extract	190 °C, 20 min	n/a	Dialysis (300 Da, Time n/a)	6–8	12.3	[51]
Bacteria identification	*Escherichia coli* (*E. coli*) *Desulfovibrio desulfuricans* (*D. desulfuricans*) *Staphylococcus sciuri* (*S. sciuri*) *Listeria monocytogenes* (*L. monocytogenes*) *Staphylococcus aureus* (*S. aureus*) *Pseudomonas aeruginosa* (*P. aeruginosa*)	3-Aminophenylboronic acid vancomycin hydrochloride polymyxin B sulfate	cis-diol (D)-Ala-(D)-Ala peptide LPS	Hydrothermal	Ammonium citrate dibasic 3-Aminophenylboronic acid vancomycin hydrochloride polymyxin B sulfate	180 °C, 4 h	3-Aminophenylboronic acid vancomycin hydrochloride polymyxin B sulfate hybridized	Centrifugation (10,000× *g* rpm, 15 min) Dialysis (1000 Da, overnight)	3–6	n/a	[118]
Antimicrobial	*Escherichia coli* *Bacillus subtilis*	n/a	n/a	Hydrothermal	Polyvinylpyrrolidone	200 °C, 6 h	N-doping	Filtration with a cylinder filtration membrane filter (0.22 μm) Centrifugation (8000× *g* rpm, 15 min)	6.5	6	[190]
					Poly (sodium- 4styrene sulfonate)		S-doping		5	9.5	
Antimicrobial	*E. coli* *S. aureus* AREC KREC	n/a	n/a	Smoking	Cigarette	Cigarette smoke of 200 cigarettes was dissolved into 1 L deionized water	n/a	Dialysis (1000 Da, Time n/a)	5.4	n/a	[191]
Antimicrobial	*S. aureus* *Bacillus subtilis* *Bacillus sp. WL-6* *E. coli* ampicillin-resistant *E. coli* *Rhizoctonia Solani* *Pyricularia Grisea*	n/a	Bacterial and fungal DNA/RNA	Electrochemical	Vitamin C	0.1 A direct current (DC) power, 3 weeks	n/a	Dialysis (500 Da, Time n/a)	5	30	[192]
Antiviral	HCoV229E	Boronic acid	S-receptor	Hydrothermal	Citric acid Ethylenediamine	Temperature: n/a, 5 h	Boronic acid functionalization (Click chemistry)	Centrifugation Dialysis (MWCO n/a, 24 h)	4.5	40	[193]
Antiviral	Japanese encephalitis Zika Dengue Porcine parvovirus Adenovirus-associated virus	n/a	n/a	Hydrothermal	Benzoxazine monomers (BZM)	180 °C, 12 h	n/a	Centrifugation (10,000× *g* rpm, 10 min) Dialysis (MWCO n/a, Time n/a)	4.4	13.1	[194]
Antiviral	Porcine reproductive Respiratory syndrome virus (PRRSV)	Glycyrrhizic acid	n/a	Hydrothermal	Glycyrrhizic acid	180 °C, 7 h	n/a	Centrifugation (10,000× *g* rpm, 10 min) Dialysis (14,000 Da, 8 h)	11.4	1.41	[195]
Antiviral	White spot syndrome virus (WSSV)	n/a	n/a	n/a	Polyamine	n/a	n/a	n/a	n/a	n/a	[196]
Antimicrobial	*Bacillus subtilis*	n/a	n/a	Reflux in concentrated nitric acid	Carbon nano-powders	Temp (°C) n/a, 48 h	EDA: EDA-CDots	Dialysis (500 Da, 48 h) Centrifugation (1000× *g*, time n/a)	4–5	20	[81]
							EPA: EPA-CDots		4–5	20	
							PEI_1200_: PEI_1200_-CDots		4–6	12	
							PEI_600_: PEI_600_-CDots		n/a	n/a	
				Hydrothermal	Citric acid PEI_1200_	n/a	PEI_1200_/CA-CDots-1 PEI_1200_/CA-CDots-2 PEI_1200_/CA-CDots-3	n/a	10	60	
Antimicrobial	*Fusarium oxysporum* *S. aureus* *P. aeruginosa* *B. subtilis* *E. coli* MCF-7 HepG2 cells	n/a	n/a	Microwave-assisted	Pomegranate extract Watermelon extract PEG 200	Microwave radiation for 2 min at the interval of 10 s each	Polymer passivation	n/a	1–5	n/a	[131]
Antibacterial	*Staphyloccocus aureus* *Escherichia coli*	Polyurethane (PU)	n/a	Swell encapsulation-shrink method (hCQDss/PU)	Pluronic F-68	ambient temperature, 48 h	n/a	n/a	n/a	n/a	[182]
Antiviral	avian leukosis virus subgroup J (ALV-J)	gp85 protein	n/a	Hydrothermal	Humic acid polytetrafluoroethylene	180 °C, 5 h	gp85 protein	Centrifugation (3000× *g* rpm, 15 min) Dialysis (1000 Da, 48 h) Centrifugation (12,000× *g* rpm, 15 min)	3–5	n/a	[197]
Antiviral	enterovirus 71 (EV71)	curcumin	ROS generation PGE_2_ production translation of EV71- and EV71-induced eIF4G cleavage phosphorylated p38 kinase	Dry-carbonization in muffle furnace	Curcumin	120 °C, 2 h (Cur-CQDs-120)	n/a	Centrifugation (35,000× *g*, 1 h) Dialysis in sodium chloride solution (0.5–1 kDa, 5 h) Dialysis in ultrapure water solution (0.5–1 kDa, 18 h)	4.2	<0.1	[198]
						150 °C, 2 h (Cur-CQDs-150)			4.5		
						180 °C, 2 h (Cur-CQDs-180)			4.8		
						210 °C, 2 h (Cur-CQDs-210)			5.2		
Antimicrobial	methicillin-resistant *Staphylococcus aureus* (MRSA) Ampicillin-resistant *Escherichia coli*	n/a	Negative charge of bacterial membranes DNA	Hydrothermal	bis-quaternary ammonium salt (BQAS)	200 °C, 12 h	N-doping	Dialysis (500 Da, 24 h)	2.15	n/a	[199]
Antimicrobial	methicillin-sensitive *Staphylococcus aureus* (ATCC 6538) methicillin-resistant *Staphylococcus aureus* (ATCC 4300) *Escherichia coli* (ATCC 25922) *Staphylococcus epidermidis* (ATCC 49134) *Bacillus cereus* (ATCC 14579)	n/a	n/a	Hydrothermal	polyethyleneimine citric acid	250 ºC, 4 h	N-doping	Dialysis (≥12,000 Da, 24 h)	70.2, 32.2, 11.5	PEI:CA ratio (1:0.5) –31 (1:1)–53 (1:2)–7.6	[138]

n/a: not available.

## Data Availability

Not applicable.
